# Molecular Insights into the Enhanced Activity and/or Thermostability of PET Hydrolase by D186 Mutations

**DOI:** 10.3390/molecules29061338

**Published:** 2024-03-17

**Authors:** Zhi Qu, Lin Zhang, Yan Sun

**Affiliations:** 1 Department of Biochemical Engineering, School of Chemical Engineering and Technology, Tianjin University, Tianjin 300350, China; qzhi@tju.edu.cn (Z.Q.); linzhang@tju.edu.cn (L.Z.); 2 Key Laboratory of Systems Bioengineering and Frontiers Science Center for Synthetic Biology (Ministry of Education), Tianjin University, Tianjin 300350, China

**Keywords:** polyethylene terephthalate, PETase, second-shell residue, thermostability, molecular mechanism

## Abstract

PETase exhibits a high degradation activity for polyethylene terephthalate (PET) plastic under moderate temperatures. However, the effect of non-active site residues in the second shell of PETase on the catalytic performance remains unclear. Herein, we proposed a crystal structure- and sequence-based strategy to identify the key non-active site residue. D186 in the second shell of PETase was found to be capable of modulating the enzyme activity and stability. The most active PETase^D186N^ improved both the activity and thermostability with an increase in *T_m_* by 8.89 °C. The PET degradation product concentrations were 1.86 and 3.69 times higher than those obtained with PETase^WT^ at 30 and 40 °C, respectively. The most stable PETase^D186V^ showed an increase in *T_m_* of 12.91 °C over PETase^WT^. Molecular dynamics (MD) simulations revealed that the D186 mutations could elevate the substrate binding free energy and change substrate binding mode, and/or rigidify the flexible Loop 10, and lock Loop 10 and Helix 6 by hydrogen bonding, leading to the enhanced activity and/or thermostability of PETase variants. This work unraveled the contribution of the key second-shell residue in PETase in influencing the enzyme activity and stability, which would benefit in the rational design of efficient and thermostable PETase.

## 1. Introduction

Plastics have become an indispensable part of daily life due to their desirable properties, such as lightweight, durability, and low price [1,2,3,4]. However, most of the plastic waste accumulates in the natural environment, exposing serious consequences for the environment and human health [5,6,7]. Polyethylene terephthalate (PET) is a semicrystalline thermoplastic composed of terephthalic acid (TPA) and ethylene glycol (EG), and is widely used in food/beverage packaging and textiles industry [8,9]. In PET, TPA and EG are linked by an ester bond that is highly resistant to biodegradation, leading to the accumulation of large amounts of PET waste in the environment [5,10]. Therefore, it is crucial to explore efficient strategies for the treatment and recycling of PET plastics. Biodegradation methods have recently received significant attention due to their low energy consumption and environmental sustainability [11].

To date, a variety of enzymes that can decompose PET have been identified, including lipases, esterases, and cutinases [12,13]. However, these enzymes generally show poor degradation activity on PET plastics with high crystallinity, and often require high temperatures to achieve the degradation of PET plastics [14,15]. Yoshida et al. isolated a bacterium (*Ideonella sakaiensis* 201-F6) from PET waste that could use PET as a carbon and energy source at moderate temperatures [16]. This bacterium can secrete a novel enzyme, PET hydrolase (PETase), which can not only degrade PET efficiently at moderate temperatures, but also shows higher activity and specificity for PET with high crystallinity than other hydrolases [4,17]. However, the degradation activity of PETase for highly crystallized PET is still low and rapidly loses enzyme activity at 40 °C, which limits the application in degrading PET waste to resolve the existing environmental crisis [17,18,19].

To overcome these limitations, various protein engineering strategies have been implemented to improve the thermostability of PETase (Table S1). For example, Son et al. applied a rational design strategy to obtain ThermoPETase, which exhibited an 8.8 °C higher *T_m_* value and a 14-fold improvement in PET degradation activity over PETase^WT^ [17]. The GRAPE strategy was developed by Wu et al. and designed the DuraPETase. The *T_m_* value and the degradation efficiency of DuraPETase against PET films were improved by 31 °C and over 300-fold, respectively [20]. The FAST-PETase, which was designed by a machine learning algorithm, exhibited an increase in *T_m_* of 18.4 °C as compared to PETase^WT^ and was able to completely degrade 51 different post-consumer PET wastes within 7 days [21]. Moreover, an automated, high-throughput directed evolution platform and a fluorescence-based high-throughput screening assay were designed for the directed evolution of PETase [15,22]. The engineered HotPETase and DepoPETase have both enhanced the robustness of the enzyme, and HotPETase could operate at the glass transition temperature of PET [15,23].

It is interesting to note that most of the mutations that enhanced the thermostability of PETase are the second-shell or distal residues (Table S1 and Figure S1). Actually, the non-active site residues can be classified into different “shells” depending on their locations in the enzyme. The second-shell residues are defined as the residues that interact with the first-shell residues [24,25], while the residues in direct contact with the substrate, cofactor, or product are the first-shell residues [24]. The residues that are located in the second shell or further and are not within van der Waals distance of any part of the substrate, cofactor, or product are distal residues [26,27]. Many works have demonstrated that non-active site residues play an important role in the regulation of enzyme functions such as activity, stability, and selectivity [25,27,28,29,30]. However, there is no research on the molecular mechanism of how the second-shell or distal residues manipulate the catalytic performance of PETase.

Therefore, the present study focused on identifying the key non-active site residue in the second shell of PETase by using a crystal structure- and sequence- (multiple sequence alignment, phylogenetic and conservative analysis) based strategy. The key non-active site residue was then mutated to the other 19 canonical amino acids to investigate how mutations of this residue manipulate the activity and stability of PETase. The structural changes of the variants were characterized by circular dichroism (CD) and fluorescence spectroscopy. Subsequently, MD simulations were performed to explore the molecular mechanisms underlying the effects of these mutations on enzymatic properties.

## 2. Results and Discussion

### 2.1. Identification of Key Non-Active Site Residue

**Figure 1:**
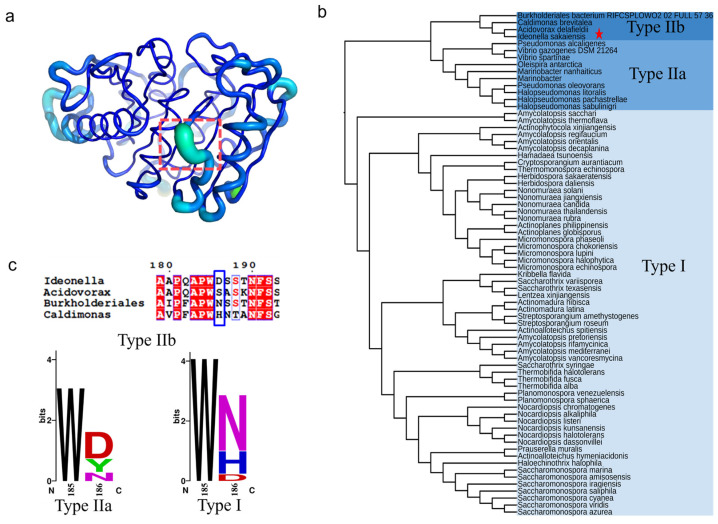
The crystal structure- and sequence-based strategy to identify the key non-active site residue in the second shell of PETase. The structure and B-factors of PETase^WT^ (**a**), where the residues D186-F191 were marked by orange dotted box, the highest B-factor region was colored in lime green and the lowest B-factor region was colored in blue. The phylogenetic tree of PETase-like enzymes (**b**), where the PETase was marked with a pentagram in the type IIb enzymes. The conservation of the D186 residue in different types of PETase-like enzymes (**c**).

The B-factor and root mean square fluctuation (RMSF) values of the β6-β7 strand connecting loop (D186-F191) were found to be significantly higher than the overall structure of PETase (Figure 1a) [17,31]. This connecting loop was one of the most flexible regions in PETase [17], a key region influencing the stability of PETase. In our previous study, we found that the D186 residue in this flexible loop could interact with the I168R and S188E mutations to form an R168-D186-E188 salt bridge network, which significantly improved the thermostability of PETase [32]. It was also reported that the mutation of D186H combined with other residues (E121 and N172) could form a water-mediated hydrogen bond [17,21], or the D186 residue interact with other mutations (I168R and S188Q) to form a hydrogen bond or a salt bridge to improve the stability of PETase [20,31]. The above analysis indicates that D186 is a key residue in the connecting loop influencing PETase stability. To further investigate the role of D186 in PETase, the amino acid sequences of PETase and 71 PETase-like enzymes from phylogenetically distinct organisms were aligned using ClustalX [33], and the phylogenetic tree of PETase-like enzymes was constructed using MEGA 11 software (Figure 1b) as reported previously [4,34]. As shown in Figure 1b, the PETase-like enzymes could be divided into types Ⅰ and Ⅱ, and the type Ⅱ enzymes could be further subdivided into two types (type Ⅱa and type Ⅱb). The type Ⅰ enzymes contained 58 PETase-like enzymes and type Ⅱ contained 14 enzymes, of which PETase belonged to type Ⅱb. Meanwhile, most of the type I and Ⅱa enzymes were reported to exhibit higher thermostability and lower PET degradation activity compared with PETase, such as the cutinase from *Thermobifida fusca* (TfCut1and TfCut2) [35,36], *Saccharomonospora viridis* (Cut190) [37], and the lipase from a metagenomic library (PET2), etc. [12,38,39], whereas the type Ⅱb enzyme variant from *Burkholderiales* bacterium (*Bur*PL^DM^) showed higher thermostability and PET degradation activity than that of PETase [40]. Thus, the conservation of the D186 residue in different types were then analyzed by the WebLogo sever (Figure 1c) [41]. This site could be amino acid D, S, N, or H in the type Ⅱb enzymes, while it could be amino acid D, Y, or N in type Ⅱa enzymes. In the type Ⅰ enzymes, the amino acid N had the most frequency, followed by H and D. The highly conserved positions determine the general commonality of all homologous enzymes, while the sequence variability and non-strictly conserved position may lead to the functional diversity, substrate, and reaction specificity of enzymes [42,43,44,45]. Based on the above analysis, it is considered that the variability of residue 186 in the different types of PETase-like enzymes was important for changing the enzymatic performance of PETase. To understand how possible mutations at this key non-active site residue in the second shell are likely to manipulate the activity and thermostability of PETase, the D186 residue was mutated to the other 19 canonical amino acids.

### 2.2. Catalytic Activity of D186 Variants

**Figure 2:**
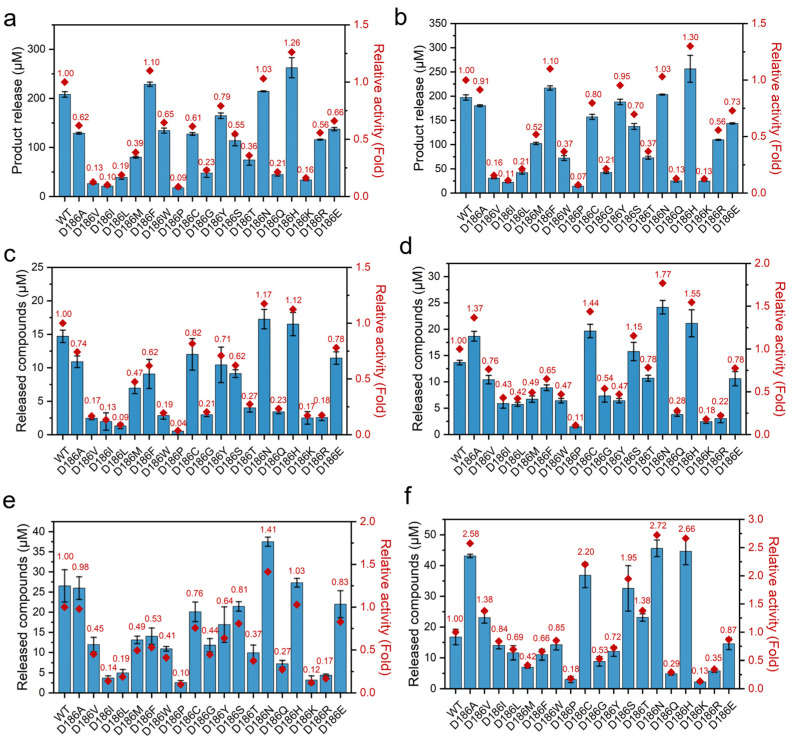
The activities of PETase^WT^ and its variants against different substrates: hydrolytic activities of PETase^WT^ and its variants at 30 °C (**a**) and 40 °C (**b**) using BHET as a substrate, where the product release was the concentration of MHET; PET film degradation activities of PETase^WT^ and its variants at 30 °C and 40 °C for 24 h (**c**,**d**) and 72 h (**e**,**f**), where the released compounds were the sum of MHET and TPA.

In order to investigate the effect of the mutations on the activity of PETase, the hydrolytic activities were tested using BHET as a substrate at 30 and 40 °C because PETase and its variants can hydrolyze BHET to MHET and EG [4,46]. As shown in Figure 2a,b, in all the nonpolar variants, PETase^D186A^, PETase^D186V^, PETase^D186I^, PETase^D186L^, PETase^D186M^, PETase^D186F^, PETase^D186W^, PETase^D186P^, and PETase^D186G^, only PETase^D186F^ showed enhanced hydrolytic activity as compared to that of PETase^WT^, and the others showed reduced activities. In the polar variants, PETase^D186C^, PETase^D186Y^, PETase^D186S^, PETase^D186T^, PETase^D186N^, and PETase^D186Q^, only PETase^D186N^ showed a 1.03-fold higher activity than PETase^WT^ at both 30 and 40 °C. In contrast, the activity of PETase^D186Q^ was significantly reduced to only 21% and 13% of that of PETase^WT^ at 30 and 40 °C, respectively. When the D186 residue was mutated to charged amino acids (H, K, R and E), PETase^D186H^ exhibited an increase in activity of 26% and 30% at 30 and 40 °C, respectively, as compared with PETase^WT^, while reduced activities were observed for PETase^D186K^, PETase^D186R^, and PETase^D186E^.

To further evaluate the effect of the mutations on PET degradation, the degradation activity against PET films (crystallinity of 28.34 ± 2.54%, Figure S2) was measured at 30 and 40 °C (Figure 2c–f). As compared with PETase^WT^, enhanced degradation activities against PET films were observed at least once at the two temperatures by PETase^D186A^, PETase^D186V^, PETase^D186C^, PETase^D186S^, PETase^D186T^, PETase^D186N^, and PETase^D186H^, while the other variants always showed reduced activities. Among these seven variants, the highest activity was obtained by PETase^D186N^ as compared with PETase^WT^ at the two temperatures. PETase^D186N^ exhibited a 1.17- and 1.41-fold increase in activity at 30 °C for 24 h and 72 h, respectively, as compared to the wild type (Figure 2c,e). This variant also showed 1.77- and 2.72-fold higher degradation activity at 40 °C for 24 h and 72 h (Figure 2d,f), respectively. This suggested that the D186N mutant could not only improve the thermostability of PETase, but also increase the PET degradation ability of PETase. However, higher activity was obtained by PETase^D186H^ for BHET. This difference further demonstrated that the hydrolysis of small molecule BHET by PETase can be different from its degradation activity on rigid PET plastics [4,47,48]. After incubation at 40 °C for 72 h, PETase^D186A^, PETase^D186V^, PETase^D186C^, PETase^D186S^, and PETase^D186T^ also showed 2.58-, 1.38-, 2.20-, 1.95-, and 1.38-fold increased activities (Figure 2f), respectively, as compared to the wild type. The above results suggest that D186 in the second shell plays a critical role in regulating the activity of PETase.

### 2.3. Thermostability of D186 Variants

**Figure 3:**
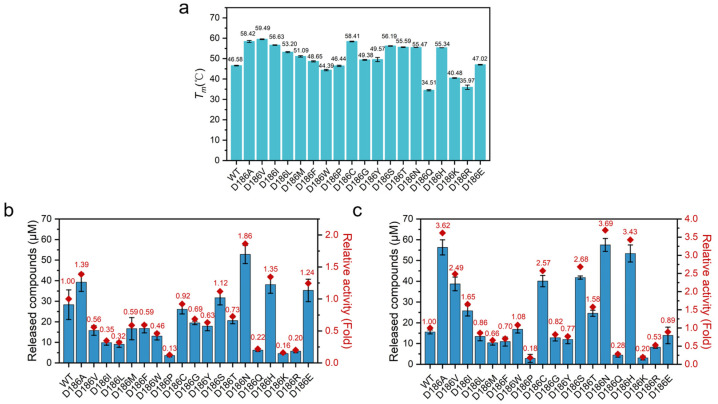
The *T_m_* and PET film degradation activities of PETase^WT^ and its variants: *T_m_* values of PETase^WT^ and its variants (**a**), PET film degradation activities of PETase^WT^ and its variants at 30 °C (**b**) and 40 °C (**c**) in 6 days, where the released compounds were the sum of MHET and TPA.

In order to evaluate how the mutations influence the stability of PETase, the *T*_m_ values of the enzymes were measured by differential scanning fluorimetry (DSF) (Figure 3a). An increase in *T_m_* values was observed with nonpolar variants except for PETase^D186W^ and PETase^D186P^. PETase^D186V^ had the highest *T_m_* value, which showed a *T_m_* value of 59.49 °C, 12.91 °C higher than that of PETase^WT^. The *T_m_* values of most polar variants also showed increases as compared with PETase^WT^. In particular, PETase^D186C^, PETase^D186S^, PETase^D186T^, and PETase^D186N^ showed increases in *T_m_* of 11.83, 9.61, 9.01, and 8.89 °C, respectively. Among the charged amino acid mutations, only PETase^D186H^ had a markedly improved *T_m_* value of 8.76 °C. These results indicated that the D186 residue could be substituted by some other amino acids to improve the thermostability of PETase, and it could manipulate the thermostability of PETase to a wide extent. Moreover, the PETase^D186N^ and PETase^D186H^ variants showed similarly higher *T_m_* values than the wild type (Figure 3a), suggesting that the differences in substrate degradation between the two variants (Figure 2) were mainly attributed to the differences in activity.

To assess the durability of PETase and its variants, the long-term degradation performance of the enzymes was then conducted at 30 and 40 °C for 6 days using PET film as a substrate. As shown in Figure 3b,c, most of the variants with improved stability (increased *T_m_* values) exhibited higher product concentrations at 40 °C than at 30 °C, whereas the variants with poor stability (decreased *T_m_* values) showed lower product concentrations at 40 °C than at 30 °C. This result further indicated that the thermostability of PETase was important for its efficient degradation of PET.

A detailed analysis of the long-term degradation experiments revealed that the degradation activity of PETase^WT^ could be maintained for 5 days at 30 °C, whereas the degradation activity was completely lost within 1 day at 40 °C (Figure S3). For most of the variants with improved thermostability, the PET degradation product concentration gradually increased at 30 °C for 6 days. Consequently, the PET degradation product concentrations of the PETase^D186N^ and PETase^D186H^ were 1.86- and 1.35-fold higher than that of PETase^WT^ after 6 days, respectively (Figure 3b). At 40 °C, the product concentrations of the PETase^D186N^ and PETase^D186H^ rapidly increased in the first 3 days (Figure S3), and the product concentrations were 3.69- and 3.43-fold higher than that of PETase^WT^ after 6 days, respectively (Figure 3c). For PETase^D186V^, the PET product concentration rapidly increased at least in the first 5 days at 40 °C (Figure S3), which was mainly due to its higher stability (Figure 3a), and the product concentration was 2.49-fold higher than that of PETase^WT^ after 6 days (Figure 3c). In addition, it was worth noting that the product concentration of PETase^D186A^ was 1.05- and 1.45-fold higher than that of PETase^D186H^ and PETase^D186V^, respectively, at 40 °C for 6 days. This phenomenon was attributed to the higher thermostability of PETase^D186A^ (*T_m_* = 58.42 °C) than PETase^D186H^ (Figure 3a) and the significantly higher activity of PETase^D186A^ than PETase^D186V^ (Figure 2). Therefore, it could be concluded that the higher product concentrations of PETase^D186N^ and PETase^D186H^ than the wild type at both 30 and 40 °C, especially for PETase^D186N^, were mainly attributed to the superimposed effects of higher activity and thermostability. Meanwhile, the higher product concentrations of PETase^D186V^ and PETase^D186A^ than the wild type were due to their improved thermostability.

Based on the results of the catalytic activity and thermostability of the D186 variants, the most active PETase^D186N^, the most stable PETase^D186V^, the stable and active PETase^D186H^ and PETase^D186A^, and the less stable and less active PETase^D186Q^ were selected for the following molecular simulation studies to unravel the molecular mechanisms.

### 2.4. Molecular Mechanism of Enhanced Catalytic Activity by D186 Mutations

**Figure 4:**
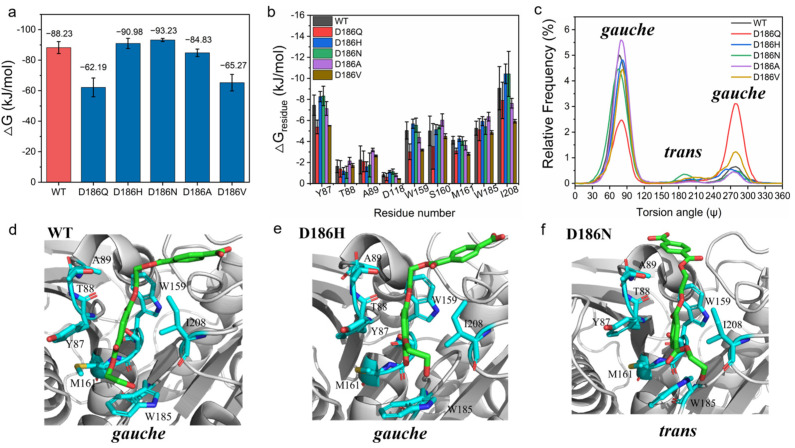
MD simulations of enzyme-2PET complexes: total binding free energies of PETase and its variants with the 2PET substrate (**a**); important residues for the 2PET-binding to PETase^WT^ and its variants (**b**); the OC-CO torsion angle Ψ in the EG units of 2PET (**c**); binding modes of the substrate 2PET into the active sites of PETase^WT^ (**d**), PETase^D186H^ (**e**), and PETase^D186N^ (**f**). The results were generated from six independent MD simulation runs.

To explore the molecular insight into the effect of D186 mutations on the catalytic activity of PETase, the substrate model 2PET was docked into PETase^WT^ and its variants [46,49], and then MD simulations were performed on enzyme-2PET complexes. From the binding free energy between the substrate and enzymes calculated using molecular mechanics Poisson−Boltzmann surface area (MM-PBSA) method (Figure 4a) [50], it was found that the total binding free energy of PETase^D186N^ (−93.23 ± 1.03 kJ·mol^−1^) and PETase^D186H^ (−90.98 ± 3.35 kJ·mol^−1^) were higher than that of PETase^WT^ (−88.23 ± 3.90 kJ·mol^−1^), whereas the total binding free energy of PETase^D186A^ (−84.83 ± 2.43 kJ·mol^−1^), PETase^D186V^ (−65.27 ± 5.41 kJ·mol^−1^) and PETase^D186Q^ (−62.19 ± 6.14 kJ·mol^−1^) were lower than that of PETase^WT^. This indicated that the D186N and D186H mutations promoted the substrate binding to the enzyme and enhanced the catalytic activity, while the D186A, D186V, and D186Q mutations were not beneficial for the substrate binding to the enzyme. Then, the residues involved in the substrate binding were identified by analyzing the contribution of each residue to the binding free energy. According to Figure 4b and Figure S4a, the residues Y87, W159, S160, M161, W185, and I208 had higher binding free energies as compared to other residues in the enzymes, indicating that these six residues were crucial for the binding of the substrate to the enzymes. This result was in good agreement with previous findings that these residues are involved in substrate binding and catalysis, and that substitution of these residues by Ala greatly reduces the catalytic activity of PETase [4,46,47,51]. Among the six residues, the binding free energies of Y87, W159, and I208 in PETase^D186H^ and PETase^D186N^ were remarkably higher than those in PETase^WT^, which could contribute to the substrate binding and increase the catalytic activity of these two variants. In contrast, the binding free energies of Y87, W159, M161, and I208 in PETase^D186Q^, PETase^D186A^ and PETase^D186V^ were lower than those in PETase^WT^, which were unfavorable for the substrate binding to the variants.

The relative frequency distribution of the catalytic distance between the hydroxyl oxygen of the catalytic serine (Ser 160) and the carbonyl carbon atom of 2PET was also calculated. As shown in Figure S4b, the catalytic distances in PETase^D186A^ (3.80 Å), PETase^D186V^ (3.80 Å), PETase^D186Q^ (3.80 Å), PETase^D186N^ (3.75 Å), and PETase^D186H^ (3.70 Å) were similar to that in PETase^WT^ (3.70 Å). So, catalytic distance change was not the cause for altering the catalytic activity of the variants. Moreover, the OC-CO torsion angle Ψ in the EG units of the substrate analysis showed that the PET chain could bind to PETase^D186N^ in the *trans* conformation with a higher probability, while the PET chain could only bind to other enzymes in the *gauche* conformation (Figure 4c–f and Figure S4c–f). Recently, it was reported that the ratio of *gauche* and *trans* conformations of PET chains was 9:1 in low crystallinity PET films [52], and that a single mutation (S238A) in the loop connecting β8 and α6 could alter the preference of PETase for the conformation of PET chains to increase the degradation activity against PET films [53]. These findings could further explain the higher activity of PETase^D186N^ as compared to PETase^D186H^ and other variants.

### 2.5. Molecular Mechanism of Enhanced Thermostability by D186 Mutations

To explore the molecular insight into the effect of D186 mutations on the thermostability of PETase, the structural changes of PETase^WT^ and its variants were analyzed. The changes in the secondary structure and tertiary structure of the enzyme variants were examined by CD and fluorescence spectroscopy (Figure S5), respectively. The secondary structure of the variants were slightly different from that of PETase^WT^ (Figure S5b), but the tertiary structure of the variants were significantly changed from that of PETase^WT^ (Figure S5c). The emission maxima (λ_max_) of PETase^D186N^, PETase^D186H^, PETase^D186A^, and PETase^D186V^ were blue shifted, in which PETase^D186V^ had the smallest λ_max_. However, the λ_max_ of PETase^D186Q^ was red shifted. This result indicated that the overall structures of PETase^D186N^, PETase^D186H^, PETase^D186A^, and PETase^D186V^ became more compact and the solvent exposure of some tryptophan and tyrosine residues was reduced, which could lead to an improvement in structural stability, whereas the overall structure of PETase^D186Q^ became looser, resulting in increased solvent exposure of some tryptophan and tyrosine residues, which could reduce its structural stability [46,54].

Subsequently, MD simulations at different temperatures (303, 313, and 403 K) were performed to analyze the overall/local structural stability and flexibility of the enzyme variants. As shown in Figures S6 and S7, the root mean square deviation (RMSD) and radius of gyration (R_g_) values of PETase^D186Q^ were obviously higher than those of PETase^WT^ at 313 and 403 K during the simulations, indicating its looser structure than the wild type, which was consistent with the results of fluorescence spectroscopy. For PETase^D186H^, PETase^D186N^, PETase^D186A^, and PETase^D186V^, the RMSD values were comparable to or lower than that of PETase^WT^. Meanwhile, the R_g_ values of PETase^D186N^, PETase^D186A^, and PETase^D186V^ were noticeably lower than that of PETase^WT^ at 403 K during the last 20 ns (Figure S7c). This suggested that the overall structural rigidity of PETase^D186H^, PETase^D186N^, PETase^D186A^, and PETase^D186V^ were stronger than that of PETase^WT^, which was also consistent with the results of fluorescence spectroscopy (Figure S5c).

**Figure 5:**
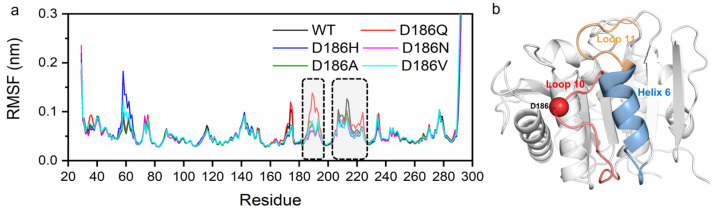
The RMSF of Cα atoms of PETase^WT^ and its variants in MD simulations at 313 K, where the regions with significant differences in average RMSF are marked in dotted boxes (**a**). Corresponding locations of the regions with notable changes of the RMSF in the structure of PETase^WT^ (**b**). Loop 10 is colored in red, Helix 6 is colored in blue, Loop 11 is colored in yellow, and the Cα atom of D186 is shown as a red sphere. The RMSF was calculated from six independent MD simulation runs.

According to the average root mean square fluctuation (RMSF) analysis of the Cα atoms of the enzyme variants (Figure 5 and Figure S8), two main regions (residues P181-P197 and residues N205-R222) of fluctuation changed in variants as compared with PETase^WT^. The average RMSF values of PETase^WT^ and its variants in these two regions were remarkably different at 313 and 403 K (Figure 3 and Figure S8b). PETase^D186H^, PETase^D186N^, PETase^D186A^, and PETase^D186V^ kept lower RMSF values than PETase^WT^ in these two regions, demonstrating that their structures were more stable than that of PETase^WT^. For PETase^D186Q^, however, the average RMSF values in these two regions were higher than those of PETase^WT^, which should be responsible for its poor thermostability. As shown in Figure 4, residue W185 of region P181-P197 and residue I208 of region N205-R222 were crucial for the binding of the substrate to the enzyme (Figure 4), and the auxiliary catalytic residue D206 located on region N205-R222 [22]. Therefore, reducing the RMSF and increasing the stability of these two regions allow the enzyme to maintain its catalytic activity at high temperatures. The analysis of mobility and structural fluctuation of PETase^WT^ and its variants by MDLovofit also confirmed the results mentioned above (Figure S9).

**Figure 6:**
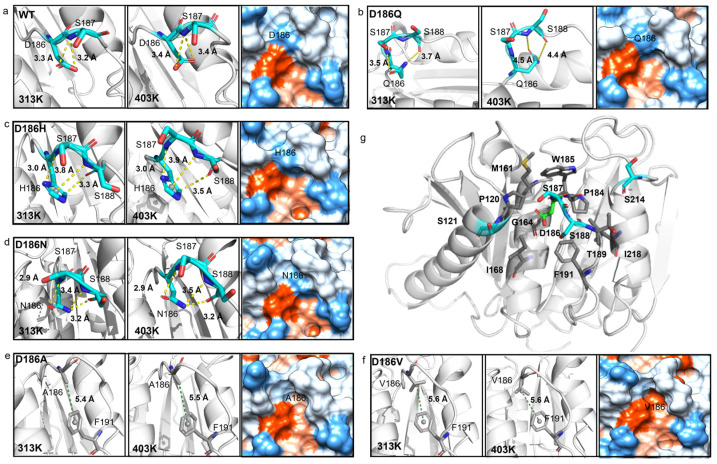
Molecular interactions between residue 186 and the surrounding residues in PETase^WT^ and its variants. PETase^WT^ (**a**), PETase^D186Q^ (**b**), PETase^D186H^ (**c**), PETase^D186N^ (**d**), PETase^D186A^ (**e**), PETase^D186V^ (**f**), and collision of polarity between D186 and the surrounding residues (5 Å) (**g**). The residue 186 and surrounding residues are shown as sticks and colored according to the elements (C: cyan/grey, N: blue, O: red, S: yellow). The protein surfaces are colored according to hydrophilicity, where the most hydrophilic are colored blue and the most hydrophobic are colored orange. Each typical structure was obtained by RMSD-based clustering analysis (cutoff = 0.075 nm) of six independent MD simulation runs.

The hydrogen bonding was then analyzed as hydrogen bonds play an important role in protein folding and thermostability [55]. It was reported that H186 could promote the water-mediated hydrogen bond formation between E121 and N172 in PETase^S121E/D186H/R280A^ [17], or H186 could form a water-mediated hydrogen-bonding network with E121 and N172 in PETase^S121E/D186H/R280A/R224Q/N233K^ [21]. The residues P181-P197, N205-S213, and S214-R222 correspond to Loop 10, Loop 11, and Helix 6 in PETase, respectively (Figure 5b). So, the interactions between residue 186 and surrounding residues were analyzed at first. For PETase^WT^, two hydrogen bonds were formed between D186 and S187 (D186_OD1_…S187_N_ and D186_OD2_…S187_N_) with the length of 3.2 Å and 3.3 Å at 313 K, respectively (Figure 6a). However, the distance/angle between the N atom of S187 and the OD1/OD2 atoms of D186 clearly increased/reduced in the last 20 ns at 403 K (Figures S10e and S11e), showing that the two hydrogen bonds were unstable in PETase^WT^ [56]. The D186Q mutation could only form a 3.5 Å hydrogen bond with S187 at 313 K and this unstable hydrogen bond rapidly cleaved at 403 K (Figure 6b, Figures S12e and S13e), indicating that this mutation enhanced the flexibility of Loop 10 and decreased the stability of PETase. In contrast, the D186H and D186N mutations introduced three stable hydrogen bonds in Loop 10, which could result in higher *T_m_* values of PETase^D186H^ and PETase^D186N^ than PETase^WT^ as revealed in the experiment (Figure 3a). The three stable hydrogen bonds in PETase^D186H^ included a hydrogen bond (H186_ND1_…S187_N_, 3.0 Å), a carbon–hydrogen bond (H186_CD2_…S188_O_, 3.3 Å), and a *π*–donor hydrogen bond (H186…S188_N_, 3.8 Å) (Figure 6c, Figures S14 and S15). For PETase^D186N^, the three stable hydrogen bonds included N186_OD1_…S187_N_ (2.9 Å), N186_ND2_…S188_O_ (3.2 Å) and N186_ND2_…S188_N_ (3.4 Å) (Figure 6d, Figures S16 and S17). Therefore, the *T_m_* value of PETase^D186H^ was similar to that of PETase^D186N^ (Figure 3a). In addition, the occupancy rates of the conventional hydrogen bonds between residue 186 and surrounding residues were also analyzed [57]. According to Tables S3–S5, the occupancy rates of the D186_OD1_…S187_N_ and D186_OD2_…S187_N_ in PETase^WT^ were similar to those of Q186_OE1_…S187_N_ and Q186_NE2_…S188_O_ in PETase^D186Q^, whereas the occupancy rates of H186_ND1_…S187_N_ in PETase^D186H^ and N186_ND2_…S188_O_ and N186_ND2_…S188_N_ in PETase^D186N^ were always higher than those of PETase^WT^ and PETase^D186Q^. Especially at 403 K, the occupancy rates of the conventional hydrogen bonds in PETase^WT^ and PETase^D186Q^ were significantly lower than those of PETase^D186H^ and PETase^D186N^. These results further indicated that these hydrogen bonds were more stable in PETase^D186H^ and PETase^D186N^ than in PETase^WT^ and PETase^D186Q^.

In the structure of PETase^WT^, there are nine hydrophobic (P120, M161, G164, I168, P184, W185, T189, F191, and I218) and four neutral (S121, S187, S188, and S214) residues within 5Å of the residue D186, which may cause D186 to conflict with the surrounding residues (Figure 6a,g). This phenomenon has been described by Son et al. as a “collision of polarity” [17]. By substituting the negatively charged residue D to the hydrophobic residue A/V, stable *π*−alkyl hydrophobic interactions formed between A186/V186 and F191 (Figure 6e,f, Figures S18 and S19), and the hydrophobic interactions between Loop 10 and its surrounding hydrophobic residues enhanced (Figure 6e,f). Furthermore, the D186A and D186V mutations changed the region from hydrophilic to hydrophobic, leading to the release of this collision of polarity (Figure 6a,e,f) and the reduction in the flexibility of Loop 10 in PETase^D186A^ and PETase^D186V^ (Figure 5a, Figures S8 and S9). Among the 20 amino acids, Val is considerably more hydrophobic than Ala, so the hydrophobic interactions were stronger in PETase^D186V^ than those in PETase^D186A^, which could lead to a higher *T_m_* of PETase^D186V^ than that of PETase^D186A^ (Figure 3a).

Next, the hydrogen bonds between Loop 10 and the two neighboring helixes, Helix 5 (residues S160-N173) and Helix 6, were analyzed in detail. Between Loop 10 and Helix 5 there were only two hydrogen bonds, W185_O_…S160_O_ and Q182_O_…S160_N_. The occupancy rates of the two hydrogen bonds were comparable as in PETase^WT^ and its variants, while only the occupancy rate of W185_O_…S160_O_ (76.4 ± 3.1%) in PETase^D186Q^ was obviously lower than PETase^WT^ (87.3 ± 2.1%) at 403 K (Table S6).

**Figure 7:**
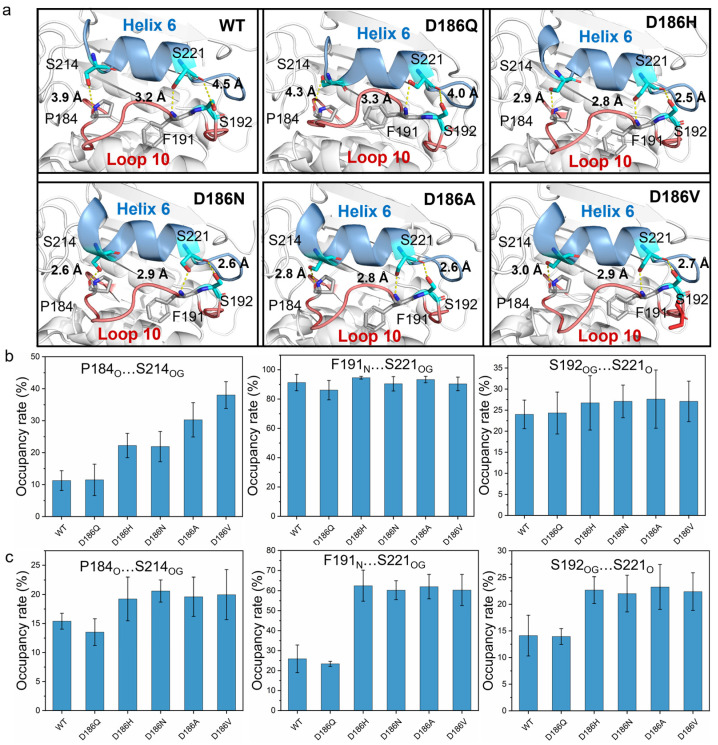
Molecular interactions between Loop 10 and Helix 6 in PETase^WT^ and its variants. The hydrogen bonds between Loop 10 and Helix 6 in PETase^WT^ and its variants determined by MD simulations (**a**). The occupancy rate of the hydrogen bonding analysis at 313 K (**b**) and 403 K (**c**), where only hydrogen bonds with occupancy rates > 10% were considered. Each typical structure was obtained by RMSD-based clustering analysis (cutoff = 0.075 nm) and the average occupancy rates were calculated from six independent MD simulation runs.

According to the typical structures obtained by RMSD-based clustering analysis (cutoff = 0.075 nm) (Figure 7a), the hydrogen bonds P184_O_…S214_OG_, F191_N_…S221_OG_, and S192_OG_…S221_O_ formed between Loop 10 and Helix 6 in PETase^D186H^, PETase^D186N^, PETase^D186A^, and PETase^D186V^. For PETase^WT^ and PETase^D186Q^, only a hydrogen bond F191_N_…S221_OG_ was observed in Loop 10 interacting with Helix 6. The occupancy rates of the hydrogen bonds F191_N_…S221_OG_ and S192_OG_…S221_O_ in PETase^WT^ and its variants were similar at temperatures below 403 K (Figure 7b and Table S7). However, the occupancy rates of P184_O_…S214_OG_ in PETase^WT^ and PETase^D186Q^ were always lower than those of the other four variants (Figure 7b,c and Table S7). At 313 K, the occupancy rates of the P184_O_…S214_OG_ in PETase^D186H^ (22.2 ± 3.8%) and PETase^D186N^ (21.9 ± 4.7%) were similar, which were lower than that of PETase^D186A^ (30.3 ± 5.4%) and PETase^D186V^ (38.0 ± 4.2%) (Figure 7b). This indicated that the more robust the hydrogen bond P184_O_…S214_OG_ was, the higher the thermostability of the variants would be (Figure 3a). Furthermore, the occupancy rates of the three hydrogen bonds in PETase^WT^ and PETase^D186Q^ were obviously lower than those of the other four variants, and PETase^D186Q^ had the lowest occupancy rates of the three hydrogen bonds at 403 K (Figure 7c). These results showed that the hydrogen bonds P184_O_…S214_OG_, F191_N_…S221_OG_, and S192_OG_…S221_O_ in PETase^D186H^, PETase^D186N^, PETase^D186A^, and PETase^D186V^ were more robust than those in PETase^WT^, whereas these hydrogen bonds in PETase^D186Q^ were more unstable than those in PETase^WT^. Therefore, locking the flexible Loop 10 and Helix 6 with more robust hydrogen bonding increased the interactions between these two regions and reduced the flexibility of the amino acid backbone, which led to the improved thermostability of the four PETase variants. In addition, the auxiliary catalytic residue D206 is located on the flexible Loop 11, and Helix 6 connects to one end of Loop 11; thus, the stabilizing effect of Helix 6 was transferred to Loop 11, leading to the flexibility decrease in the loop (Figure 5 and Figure S8b). The flexibility decrease (or rigidity increase) of Loop 11 could stabilize the auxiliary catalytic residue D206, and further enhance the thermostability of the variants [22].

## 3. Materials and Methods

### 3.1. General Information

Bis-2(hydroxyethyl) terephthalate (BHET) and terephthalic acid (TPA) of analytical grade were purchased from Aladdin (Shanghai, China). Mono(2-hydroxyethyl) terephthalate (MHET) of analytical grade was purchased from MOLBASE (Shanghai, China). PET films (transparent, 0.25 mm thickness) were obtained from Goodfellow (Cambridge, England). All other chemicals and reagents of at least reagent grade were purchased from commercial sources and used without further purification unless otherwise stated. TransStart^®^ FastPfu DNA polymerase, DMT enzyme, *pEASY*^®^-Basic Seamless Cloning and Assembly Kit were purchased from TransGen Biotech (Beijing, China). *Escherichia coli* (*E. coli*) BL21 (DE3) and expression vector pET-22b (+) were obtained from Solarbio (Beijing, China). The primer synthesis and DNA sequencing analysis were conducted by GENEWIZ and Tsingke (Tianjin, China).

### 3.2. Site-Directed Mutagenesis, Protein Expression, and Purification

The gene encoding PETase from *Ideonella sakaiensis* 201-F6 (GenBank accession number: GAP38373.1) was commercially synthesized by GENEWIZ (Beijing, China), and then subcloned into pET-22b (+) vector between *Nde* I and *Xho* I restriction sites. The final construct vector pET-22b: PETase was transformed into *E. coli* BL21 (DE3).

The site-directed mutagenesis was constructed by PCR using a recombinant plasmid pET22b (+) containing the PETase gene as a template. The PCR procedure was as follows: 95 °C for 2 min, (95 °C for 20 s, 56 °C for 20 s, 72 °C for 3.5 min) with 35 cycles and extension at 72 °C for 5 min. The PCR reaction mixture contained 32 μL distilled water, 10 μL 5× buffer, 4 μL 2.5 mM dNTPs, 1 μL each forward and reverse primer (10 μM), 1 μL recombinant plasmid, and 1 μL TransStart^®^FastPfu DNA polymerase (TransGen Biotech, Beijing, China). The PCR products were digested with 1 μL DMT enzyme at 37 °C for 1 h and then separately transformed into *E. coli* BL21 (DE3) cells for protein expression. Residue D186 was substituted by the other 19 canonical amino acids by this method (Table S2), and the sequences were verified by DNA sequencing.

A single colony of wild type or variants was picked and incubated in a 5 mL LB medium containing 100 μg·mL^−1^ ampicillin at 37 °C and 220 rpm for 12 h. Then, 2 mL of the preculture cells were inoculated into 200 mL of LB medium containing 100 μg mL^−1^ ampicillin at 37 °C and 220 rpm. When the optical density at 600 nm was between 0.8 and 1.0, 0.5 mM isopropyl-β-D-1-thiogalactoside (IPTG) was added to the LB medium. The cultures were then incubated for another 24 h at 16 °C and 160 rpm for protein overexpression. The cells were harvested by centrifugation (5000× *g*, 30 min) at 4 °C, and the cell pellet was resuspended in lysis buffer (50 mM Na_2_HPO_4_-HCl, 100 mM NaCl, 20 mM imidazole, pH 7.0). 

The resuspended cells were disrupted by ultrasonication in an ice-water bath for 30 min. Insoluble cell debris was removed by centrifugation (12,000× *g*, 30 min, 4 °C) and filtrated through a 0.45 μm syringe filter. The supernatant was loaded onto a 5 mL Ni-NTA FF column (GE Healthcare, Solingen, Germany), and the affinity column was pre-equilibrated with lysis buffer. Non-specific adsorbed proteins were removed by washing with lysis buffer, and the target enzyme was eluted with elution buffer (50 mM Na_2_HPO_4_-HCl, 100 mM NaCl, 300 mM imidazole, pH 7.0). The protein fractions were then transferred into desalting buffer (50 mM Na_2_HPO_4_-HCl, 100 mM NaCl, pH 7.0) by size-exclusion chromatography on an AKTA Basic system with a Superdex200 Increase 10_300 GL column (GE Healthcare, Germany). All purification steps were conducted at 4 °C. The purified enzyme was checked by SDS-PAGE, and the concentration of the enzyme was quantified by BCA Protein Assay Kit (Solarbio, Beijing, China).

### 3.3. Enzyme Activity Assay for BHET and PET Film Degradation

The activities of the wild-type PETase and its variants were first determined at 30 and 40 °C by monitoring the hydrolysis of BHET to MHET and EG using a high-performance liquid chromatography (HPLC) system (1100 Series HPLC, Agilent, Santa Clara, CA, USA), as reported previously [46,58]. The reaction mixture contained 910 μL reaction buffer (80 mM Na_2_HPO_4_−HCl, 40 mM NaCl, pH 7.0), 80 μL BHET substrate (2.5 g·L^−1^), and 10 μL enzyme solution (5 μM). The enzyme reaction was terminated by adding an equal volume of 160 mM phosphoric acid solution (20% (*v*/*v*) DMSO, pH 2.5) and heating at 85 °C for 10 min. Then, the reaction mixture was centrifuged at 13,000× *g* for 10 min, and the supernatant was filtered through a 0.22 μm membrane for HPLC analysis. Enzyme activity was defined as the concentration (μM) of MHET released by enzyme (50 nM) catalyzed degradation of the BHET substrate (200 mg·L^−1^) for 30 min, as previously reported [46,58].

The PET film was used as a substrate to determine the degradation activity of PET by the wild-type PETase and its variants. The PET film has a crystallinity of 28.34 ± 2.54% (Figure S1) and was cut into a circular form with a diameter of 6 mm. The PET film was soaked with 12.5 μL of enzyme (5 μM) in 300 μL of glycine-NaOH buffer (50 mM, pH 9.0) at 30 and 40 °C for 1 to 6 days. After removing the PET film from the reaction mixture, the reaction was terminated by adding an equal volume of 160 mM phosphoric acid solution (20% (*v*/*v*) DMSO, pH 2.5) and heating at 85 °C for 10 min. Then, the reaction mixture was centrifuged at 13,000× *g* for 10 min. The supernatant was filtered through a 0.22 μm membrane and analyzed by HPLC.

### 3.4. Assay of Enzyme Thermostability 

To analyze the thermostability of the wild-type PETase and its variants, the melting temperature (*T_m_*) was determined by differential scanning fluorimetry (DSF). The concentration of purified protein was diluted to 5 μM with desalting buffer (50 mM Na_2_HPO_4_-HCl, 100 mM NaCl, pH 7.0). The SYPRO Orange dye 5000× (Sigma-Aldrich, Shanghai, China) was diluted to 100× with pure water. The diluted protein solution (20 μL) was mixed with 5 μL of diluted SYPRO Orange dye and loaded onto a 96-well PCR plate (Roche, Shanghai, China). DSF experiments were performed using a Light Cyder480 real-time PCR system (Roche, Santa Clara, CA, USA). The 96-well PCR plate was heated from 25 to 100 °C at a rate of 1.8 °C·min^−1^. The excitation and emission wavelengths were set at 465 nm and 580 nm, respectively. A single fluorescence measurement was taken every 0.03 s. The apparent *T_m_* was determined from the first derivative curve.

### 3.5. CD and Fluorescence Spectroscopy

CD spectroscopy was used to assess the second structure of the wild-type PETase and its variants. The concentration of protein was diluted to 0.143 mg·mL^−1^ with desalting buffer. CD studies were then carried out using the J-810 CD spectrometer (JASCO, Tokyo, Japan) in a 1.0 mm quartz cell at 25 °C. The spectra were recorded at a far-UV wavelength from 190 to 260 nm with a bandwidth of 1 nm at a speed of 100 nm·min^−1^. The spectra of the desalting buffer were subtracted as background.

Fluorescence spectroscopy was used to determine the conformational change in the wild-type PETase and its variants. The concentration of the sample was maintained at 3 μM. The intrinsic fluorescence spectra were performed on a luminescence spectrometer (PerkinElmer, Shelton, CT, USA) with a 1 cm quartz cell at 25 °C. The excitation wavelength was set at 280 nm, and the emission spectra were recorded from 285 nm to 450 nm. The slit widths of excitation and emission were both set to 5.0 nm, and the scanning speed of emission spectra was set to 200 nm·min^−1^ [59].

### 3.6. Molecular Docking and Molecular Dynamics (MD) Simulations

The three-dimensional structure of the wild-type PETase was obtained from the Protein Data Bank (PDB ID: 6EQE) [60]. The structure of variants (D186Q, D186H, D186N, D186A, D186V) was constructed by the mutate module of Pymol software (https://pymol.org/ (accessed on 1 October 2023), with the structure of the wild-type PETase as a template. The MD simulations of wild type and variants were performed using GROMACS 5.1.4 software with the AMBER99SB-ILDN force field [61,62,63]. The enzymes were placed in the center of a cubic box at a distance of 10 Å from the edge of the box. The six systems were then solvated with the TIP3P water model and Cl^-^ ions were added to keep all the systems neutral. All the systems were minimized for 50,000 steps until the maximum force reached 1000 kJ·mol^−1^·nm^−1^ using the steepest descent algorithm. Then, the systems were heated from 0 K to 303 K through v-rescal under NVT ensemble for 100 ps with a time-step of 2 fs. Subsequently, the systems were equilibrated for 100 ps under NPT ensemble at the constant temperature of 303 K and pressure of 1.0 atm using the Parrinello–Rahman thermostat. After this, the restraints for the protein were removed and six independent 50 ns MD simulations were performed for each system under NPT ensemble. The MD simulations for the wild-type PETase and its variants at 313 K and 403 K were performed using the same protocol as that at 303 K.

The typical structures obtained from the above simulations were used for subsequent docking. The model substrate, consisting of two repeating units of ethylene terephthalate (2PET) [46,49], was docked into the wild-type PETase and its variants by Autodock [64]. The MD simulations for the enzyme-substrate complexes were performed at 303 K for 50 ns, using the same protocol as that described above. Six independent simulations were performed for each system.

The linear constraint solver (LINCS) algorithm was used to constrain the bonds. The particle-mesh Ewald (PME) algorithm was used to calculate the electrostatic interactions. The cutoffs of the neighboring atom list, Lennard-Jones (LJ) potential and Coulomb potential energies were all set as 12 Å. The trajectories analysis were performed using the Gromacs package and VMD 1.9.3 software [65].

### 3.7. Measurement of PET Crystallinity

The percentage of crystallinity (X_c_) for PET film was analyzed by the differential scanning calorimetry (DSC) instrument (Q20, TA instrument, New Castle, DE, USA) with the methods reported previously [17,46,66]. Approximately 2–3 mg of the PET sample was cooled to 0 °C and equilibrated for 1 min. Then, the sample was heated to 300 °C (10 °C·min^−1^) and maintained at 300 °C for 1 min. The sample was then cooled from 300 °C to 0 °C (10 °C·min^−1^). The percentage of crystallinity was calculated using the equation:

(1) $$X_{c}\left(\%\right)=\left[\frac{\Delta H_{m}-\Delta H_{c}}{\Delta H_{f}}\right]\times 100$$

where $\Delta H_{m}$ is the value of melting enthalpy (J·g^−1^), $\Delta H_{c}$ is the value of cold crystallization (J·g^−1^), and $\Delta H_{f}$ is the melting enthalpy of 100% crystalline PET (140.1 J·g^−1^).

### 3.8. HPLC Analysis 

The supernatant of reaction solution (20 μL) was analyzed by an Agilent 1100 series LC system (Agilent, USA) equipped with an Ultimate XB-C18 column (4.6 × 250 mm, 5 μm, Welch Materials, Shanghai, China) at 25 °C. The mobile phase consisted of 70% (*v*/*v*) distilled water, 20% (*v*/*v*) acetonitrile, and 10% (*v*/*v*) formic acid. The flow rate was set at 1.0 mL·min^−1^, and the detection wavelength for aromatic products (BHET, MHET, and TPA) was set at 254 nm.

## 4. Conclusions

In the present study, a non-active key amino acid residue in the second shell, D186, was identified using a crystal structure- and sequence-based strategy. The effects of this residue on the catalytic activity and thermostability of PETase were investigated by mutating D186 to the other 19 canonical amino acids. Among the variants, the *T_m_* values of PETase^D186V^ and PETase^D186A^ increased by 12.91 and 11.84 °C, respectively, and their degradation efficiency of PET were 2.49 and 3.62 times higher than that of PETase^WT^ at 40 °C. PETase^D186N^ and PETase^D186H^ showed both improved thermostability and activity in PET degradation. PETase^D186N^ had the highest degradation efficiency against PET, being 1.86 and 3.69 times higher than the wild type at 30 and 40 °C, respectively. These results indicated that this key non-active amino acid was important for modulating the catalytic activity and thermostability of PETase. The structural analysis and MD simulations revealed that the D186 mutations (1) altered the tertiary structure rather than the secondary structure of the enzyme molecule, (2) elevated the binding free energy of the substrate to the enzyme and changed the substrate binding mode, resulting in the increase in catalytic activity of the enzyme, and/or (3) reduced the flexibility of Loop 10 mainly by hydrogen bonding and hydrophobic interactions, and locked flexible Loop 10 and Helix 6 by robust hydrogen bonding, leading to the enhanced enzyme thermostability. It is anticipated that this finding can be applied as a rational design strategy to improve thermostability of PET hydrolases and other enzymes, and it suggests that other non-active site residues in the second shell may be targeted as hot spots to further improve the catalytic activity and thermostability of PETase.

## Data Availability

Data are available on request from the authors.

## Supplementary Materials

The following supporting information can be downloaded at: https://www.mdpi.com/article/10.3390/molecules29061338/s1, Table S1: Engineered PETases for improved thermostability [15,17,20,21,22,31,67,68,69]. Table S2: The sequences of primers for site-directed mutation. Table S3: The occupancy rates of the conventional hydrogen bonds between residue 186 and surrounding residues at 303 K. Table S4: The occupancy rates of the conventional hydrogen bonds between residue 186 and surrounding residues at 313 K. Table S5: The occupancy rates of the conventional hydrogen bonds between residue 186 and surrounding residues at 403 K. Table S6: The occupancy rates of the hydrogen bonds between Loop 10 and Helix 5. Table S7: The occupancy rates of the hydrogen bonds between Loop 10 and Helix 6. Figure S1: Spatial distribution of point mutations that influence the thermostability of PETase. Figure S2: The differential scanning calorimetry (DSC) spectrum of PET film. Figure S3: PET film degradation activities of PETase^WT^ and its variants at 30 °C and 40 °C for 6 days. Figure S4: Molecular dynamics (MD) simulations of enzyme-2PET complexes. Figure S5: The crystal structure of PETase, the circular dichroism spectra and the intrinsic fluorescence spectra of PETase^WT^ and its variants at 25 °C. Figure S6: Root mean square deviation (RMSD) of the Cα atoms of PETase^WT^ and its variants in the MD simulations. Figure S7: Radius of gyration (R_g_) of the Cα atoms of PETase^WT^ and its variants in the MD simulations. Figure S8: Root mean square fluctuations (RMSF) of the Cα atoms of PETase^WT^ and its variants in the MD simulations. Figure S9: The mobility and structural fluctuations of the Cα atoms of PETase^WT^ and its variants obtained with MDLovofit. Figure S10: The distance between the atom OD1 or OD2 of D186 and the atom N of S187, and the relative frequency of these two distances in PETase^WT^ during the MD simulations. Figure S11: The angle between the acceptor atom (D) of D186_OD1_ or D186_OD2_, the hydrogen atom (H), and the donor atom (A) of S187_N_, and the relative frequency of these two angles in PETase^WT^ during the MD simulations [56]. Figure S12: The distance between the atom OE1 of Q186 and the atom N of S187, the distance between the atom NE2 of Q186 and the atom O of S188, and the relative frequency of these two distances in PETase^D186Q^ during the MD simulations. Figure S13: The angle between the acceptor atom (A) of Q186_OE1_, the hydrogen atom (H), and the donor atom (D) of S187_N_, the angle between the acceptor atom (A) of S186_O_, the hydrogen atom (H), and the donor atom (D) of S186_NE2_, and the relative frequency of these two angles in PETase^D186Q^ during the MD simulations [56]. Figure S14: The distance between the atom ND1 of H186 and the atom N of S187, the distance between the atom CD2 of H186 and the atom O of S188, the distance between the centroid of imidazole ring of H186 and the N atom of S188, and the relative frequency of these three distances in PETase^D186H^ during the MD simulations. Figure S15: The angle between the acceptor atom (A) of H186_ND1_, the hydrogen atom (H), and the donor atom (D) of S187_N_, the angle between the acceptor atom (A) of S186_O_, the hydrogen atom (H), and the donor atom (D) of H186_CD2_, and the relative frequency of these two angles in PETase^D186H^ during the MD simulations [56]. Figure S16: The distance between the atom OD1 of N186 and the atom N of S187, the distance between the atom ND2 of N186 and the atom O of S188, the distance between the atom ND2 of N186 and the atom N of S188, and the relative frequency of these two distances in PETase^D186N^ during the MD simulations. Figure S17: The angle between the acceptor atom (A) of N186_OD1_, the hydrogen atom (H), and the donor atom (D) of S187_N_, the angles between the donor atom (D) of N186_ND2_, the hydrogen atom (H), and the acceptor atom (A) of S188_O_ or S188_N_, and the relative frequency of these three angles in PETase^D186N^ during the MD simulations [56]. Figure S18: The distance between the centroid of alkyl of A186 and π ring of F191, and the relative frequency of this distance in PETase^D186A^ during the MD simulations. Figure S19: The distance between the centroid of alkyl of V186 and π ring of F191, and the relative frequency of this distance in PETase^D186V^ during the MD simulations.
