# Supplementary material for: Molecular Insights into the Enhanced Activity and/or Thermostability of PET Hydrolase by D186 Mutations
Source: Molecules. 2024 Mar 17;29(6):1338. doi: 10.3390/molecules29061338 (PMC10975908; doi:10.3390/molecules29061338)
Supplement: Supplementary file 1 [file molecules-29-01338-s001.zip › molecules-2916373-supplementary.pdf]

Supplementary materials

Molecular Insights into the Enhanced Activity and/or Thermostability of PET Hydrolase by D186 Mutations

Zhi Qu [1], Lin Zhang [1,2] and Yan Sun [1,2,*]

[1] *Department of Biochemical Engineering, School of Chemical Engineering and Technology, Tianjin University, Tianjin 300350, China*

[2] *Key Laboratory of Systems Bioengineering and Frontiers Science Center for Synthetic Biology (Ministry of Education), Tianjin University, Tianjin 300350, China*

* Corresponding author:

E-mail address: ysun@tju.edu.cn (Y. Sun)

Table S1. Engineered PETases for improved thermostability.

Enzyme	T_m value	Design approach	Refs
PETase (W159H/F229Y)	T_m = 61.2 °C	A web tool Premuse	[1]
ThermoPETase (S121E/D186H/R280A)	T_m = 57.6 °C	Structure-based design	[2]
TS-PETase (S121E/D186H/R280A/N233C/S282C)	T_m = 69.4 °C	Structural comparison with other homologous PET hydrolases	[3]
S92R/D157E/R251A	T_m = 54.80 °C	Electrostatic interaction-based strategy	[4]
DuraPETase (S214H/I168R/W159H/S188Q/R280A/A180I/G165A/Q119Y/L117F/T140D)	T_m = 77.0 °C	GRAPE strategy	[5]
DuraPETase-2M (DuraPETase + N233C/S282C)	T_m = 81.1 °C	Structural and sequence comparison with homologous PET hydrolases	[6]
FAST-PETase (S121E/D186H/R280A/R224Q/N233K)	T_m = 67.1 °C	Machine learning algorithm	[7]
HotPETase (S58A/S61V/R90T/K95N/Q119K/S121E/M154G/P181V/Q182M/D186H/S207R/N212K/S213E/S214Y/R224L/N233C/N241C/K252M/T270Q/R280A/S282C)	T_m = 82.5 °C	Directed evolution	[8]

DepoPETase (T88I/Q119R/D186H/D220N/N233K/N246D/R260Y/S290P)	T_m = 69.4°C	Directed evolution	[9]

Table S2. The sequences of primers for site-directed mutation.

Mutants	Primers	
	Forward	Reverse
D186A	GCCCCATGGGCTAGCAGCA CCAATTTCAGCAGC	GGTGCTGCTAGCCCATGGGGC TTGTGGCGCCGC
D186G	GCCCCATGGGGTAGCAGCA CCAATTTCAGCAGC	GGTGCTGCTACCCCATGGGGC TTGTGGCGCCGC
D186V	GCCCCATGGGTTAGCAGCA CCAATTTCAGCAGC	GGTGCTGCTAACCCATGGGGC TTGTGGCGCCGC
D186L	GCCCCATGGCTTAGCAGCA CCAATTTCAGCAGC	GGTGCTGCTAAGCCATGGGGC TTGTGGCGCCGC
D186I	GCCCCATGGATTAGCAGCA CCAATTTCAGCAGC	GGTGCTGCTAATCCATGGGGC TTGTGGCGCCGC
D186S	GCCCCATGGTCTAGCAGCA CCAATTTCAGCAGC	GGTGCTGCTAGACCATGGGGC TTGTGGCGCCGC
D186T	GCCCCATGGACTAGCAGCA CCAATTTCAGCAGC	GGTGCTGCTAGTCCATGGGGC TTGTGGCGCCGC
D186M	GCCCCATGGATGAGCAGCA CCAATTTCAGCAGC	GGTGCTGCTCATCCATGGGGC TTGTGGCGCCGC
D186E	GCCCCATGGGAAAGCAGC ACCAATTTCAGCAGC	GGTGCTGCTTTCCCATGGGGC TTGTGGCGCCGC
D186N	GCCCCATGGAATAGCAGCA CCAATTTCAGCAGC	GGTGCTGCTATTCCATGGGGC TTGTGGCGCCGC
D186Q	GCCCCATGGCAAAGCAGCA CCAATTTCAGCAGC	GGTGCTGCTTTGCCATGGGGC TTGTGGCGCCGC
D186K	GCCCCATGGAAGAGCAGC ACCAATTTCAGCAGC	GGTGCTGCTCTTCCATGGGGC TTGTGGCGCCGC
D186R	GCCCCATGGCGTAGCAGCA CCAATTTCAGCAGC	GGTGCTGCTACGCCATGGGGC TTGTGGCGCCGC
D186H	GCCCCATGGCATAGCAGCA CCAATTTCAGCAGC	GGTGCTGCTATGCCATGGGGC TTGTGGCGCCGC
D186P	GCCCCATGGCCTAGCAGCA CCAATTTCAGCAGC	GGTGCTGCTAGGCCATGGGGC TTGTGGCGCCGC

D186F	GCCCCATGGTTCAGCAGCACCAATTTCAGCAGC	GGTGCTGCTGAACCATGGGGCTTGTGGCGCCGC
D186Y	GCCCCATGGTATAGCAGCACCAATTTCAGCAGC	GGTGCTGCTATACCATGGGGCTTGTGGCGCCGC
D186W	GCCCCATGGTGGAGCAGCACCAATTTCAGCAGC	GGTGCTGCTCCACCATGGGGCTTGTGGCGCCGC
D186C	GCCCCATGGTGTAGCAGCACCAATTTCAGCAGC	GGTGCTGCTACACCATGGGGCTTGTGGCGCCGC

Table S3. The occupancy rates of the conventional hydrogen bonds between residue 186 and surrounding residues at 303K.

Enzyme	Hydrogen bond	Occupancy rate
WT	$D186_{OD1}$…$S187_{N}$	37.2 ± 5.9
	$D186_{OD2}$…$S187_{N}$	35.7 ± 6.7
D186Q	$Q186_{OE1}$…$S187_{N}$	45.2 ± 3.6
	$Q186_{NE2}$…$S188_{O}$	21.2 ± 5.2
D186H	$H186_{ND1}$…$S187_{N}$	59.4 ± 2.0
D186N	$N186_{OD1}$…$S187_{N}$	19.1 ± 1.2
	$N186_{ND2}$…$S188_{O}$	84.4 ± 4.2
	$N186_{ND2}$…$S188_{N}$	62.5 ± 1.7

Table S4. The occupancy rates of the conventional hydrogen bonds between residue 186 and surrounding residues at 313K.

Enzyme	Hydrogen bond	Occupancy rate
WT	$D186_{OD1}$…$S187_{N}$	48.0 ± 2.0
	$D186_{OD2}$…$S187_{N}$	25.6 ± 6.9
D186Q	$Q186_{OE1}$…$S187_{N}$	47.6 ± 3.2
	$Q186_{NE2}$…$S188_{O}$	28.9 ± 5.0
D186H	$H186_{ND1}$…$S187_{N}$	57.2 ± 1.6
D186N	$N186_{OD1}$…$S187_{N}$	19.6 ± 1.0
	$N186_{ND2}$…$S188_{O}$	84.6 ± 5.1
	$N186_{ND2}$…$S188_{N}$	61.6 ± 1.4

Table S5. The occupancy rates of the conventional hydrogen bonds between residue 186 and surrounding residues at 403K.

Enzyme	Hydrogen bond	Occupancy rate
WT	$D186_{OD1}...S187_{N}$	12.7 ± 3.6
	$D186_{OD2}...S187_{N}$	11.4 ± 5.6
D186Q	$Q186_{OE1}...S187_{N}$	11.5 ± 1.6
	$Q186_{NE2}...S188_{O}$	6.7 ± 2.1
D186H	$H186_{ND1}...S187_{N}$	49.6 ± 6.9
D186N	$N186_{OD1}...S187_{N}$	15.0 ± 3.4
	$N186_{ND2}...S188_{O}$	31.9 ± 3.0
	$N186_{ND2}...S188_{N}$	27.1 ± 1.8

Table S6. The occupancy rates of the hydrogen bonds between Loop 10 and Helix 5.

Temperature	Enzyme	$W185_N$…$S160_O$ (%)	$S160_N$…$Q182_O$ (%)
303 K	WT	93.7 ± 1.1	68.9 ± 4.5
	D186Q	93.3 ± 1.6	62.5 ± 3.6
	D186H	90.8 ± 3.6	66.7 ± 9.6
	D186N	91.7 ± 4.4	73.0 ± 6.3
	D186A	94.3 ± 1.2	67.1 ± 8.1
	D186V	95.0 ± 3.2	72.1 ± 8.1
313 K	WT	93.7 ± 0.4	69.1 ± 3.9
	D186Q	91.5 ± 2.4	65.3 ± 3.0
	D186H	93.5 ± 1.8	66.8 ± 7.1
	D186N	92.4 ± 1.6	67.9 ± 3.7
	D186A	94.1 ± 0.7	66.1 ± 8.8
	D186V	95.5 ± 0.7	65.9 ± 2.6
403 K	WT	87.3 ± 2.1	66.6 ± 3.5
	D186Q	76.4 ± 3.1	66.1 ± 3.2
	D186H	84.8 ± 3.8	66.1 ± 3.9
	D186N	84.0 ± 5.8	66.2 ± 3.4
	D186A	87.4 ± 3.1	67.7 ± 2.0
	D186V	85.9 ± 2.6	65.2 ± 2.4

Table S7. The occupancy rates of the hydrogen bonds between Loop 10 and Helix 6.

Temperature	Enzyme	$F191_{N}...S221_{OG}$ (%)	$S192_{OG}...S221_{O}$ (%)	$S214_{OG}...P184_{O}$ (%)
303 K	WT	92.7 ± 4.4	24.6 ± 5.5	15.0 ± 5.7
	D186Q	91.8 ± 6.4	24.6 ± 2.9	11.8 ± 4.5
	D186H	91.2 ± 7.2	24.5 ± 5.3	25.9 ± 5.1
	D186N	93.4 ± 1.7	23.5 ± 1.0	24.5 ± 9.5
	D186A	92.1 ± 2.4	24.5 ± 5.9	26.2 ± 3.5
	D186V	92.0 ± 3.3	19.4 ± 3.5	39.0 ± 5.2
313 K	WT	91.3 ± 5.6	24.0 ± 3.4	11.3 ± 3.1
	D186Q	86.1 ± 6.6	24.3 ± 5.0	11.5 ± 4.9
	D186H	94.6 ± 1.0	26.7 ± 6.5	22.2 ± 3.8
	D186N	90.5 ± 4.90	27.1 ± 3.9	21.9 ± 4.7
	D186A	93.3 ± 2.2	27.6 ± 6.9	30.3 ± 5.4
	D186V	90.4 ± 4.6	27.1 ± 4.8	38.0 ± 4.2
403 K	WT	26.0 ± 6.9	14.1 ± 3.8	15.4 ± 1.4
	D186Q	23.4 ± 1.3	14.0 ± 1.5	13.5 ± 2.3
	D186H	62.5 ± 7.7	22.7 ± 2.5	19.2 ± 3.8
	D186N	60.3 ± 4.8	22.0 ± 3.4	20.6 ± 1.9
	D186A	62.0 ± 6.1	23.2 ± 4.2	19.6 ± 3.4
	D186V	60.3 ± 7.8	22.4 ± 3.5	20.0 ± 4.3

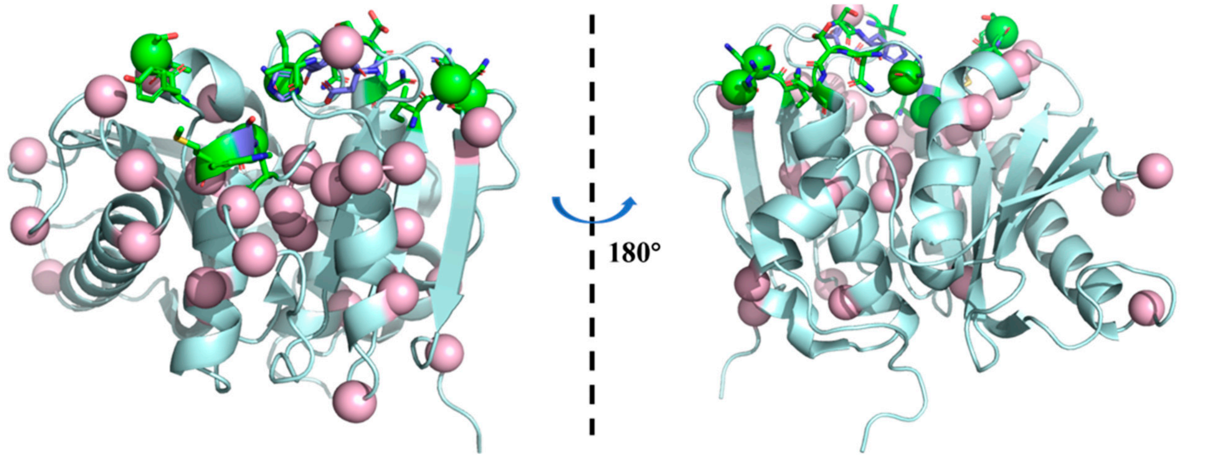

Figure S1. Spatial distribution of point mutations that influence the thermostability of PETase. The catalytic triad and residues involved in substrate binding (the first-shell residues) are shown as blue and green sticks, respectively. The mutated residues in the first shell are shown as green spheres, and the mutated residues in the second shell or distal sites are shown as lightpink spheres. The data for the mutation sites are based on Table S1.

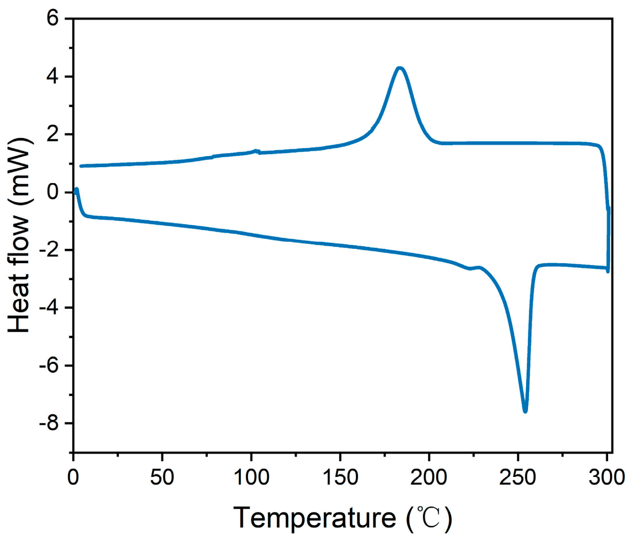

Figure S2. The differential scanning calorimetry (DSC) spectrum of PET film.

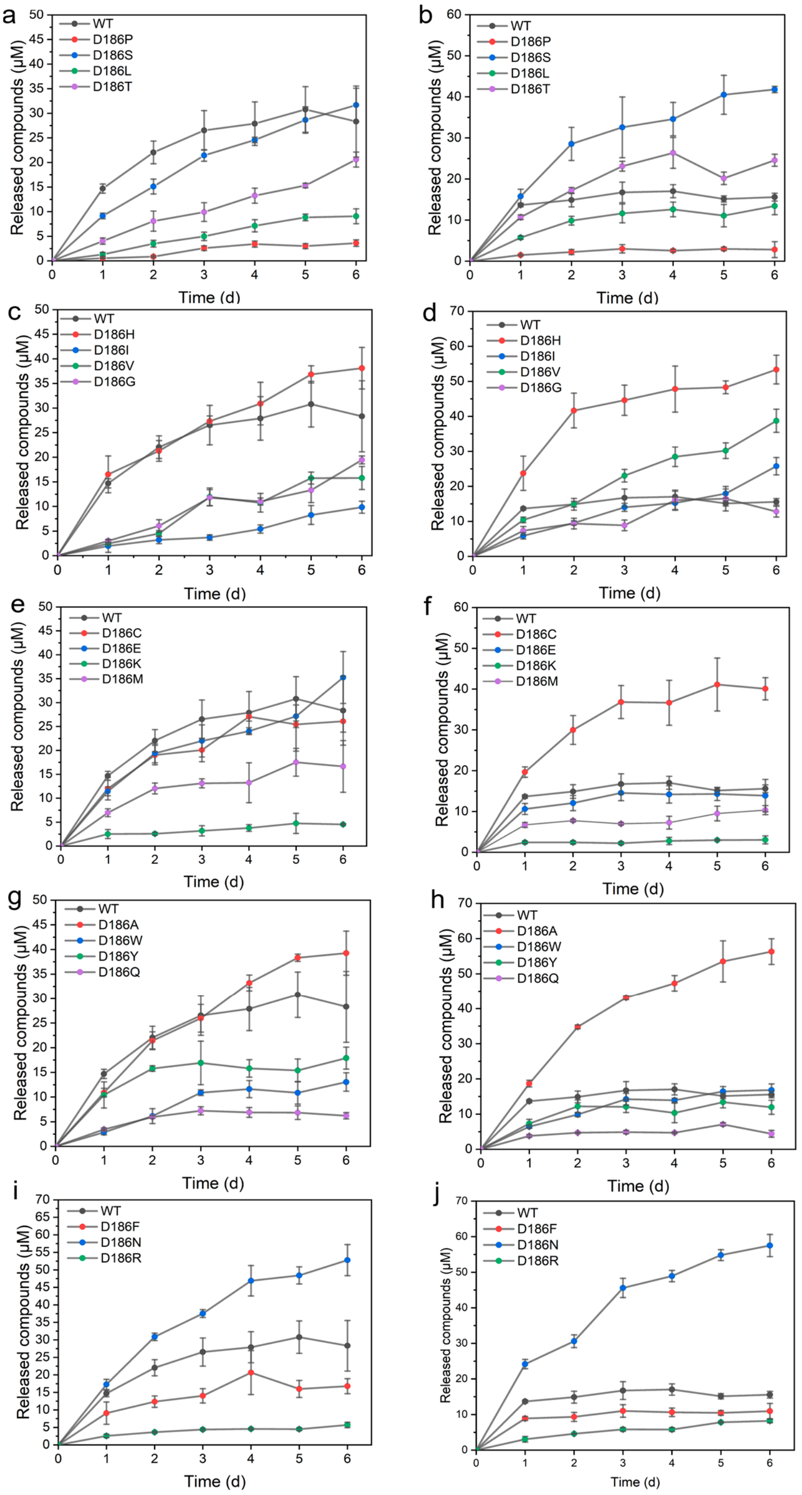

Figure S3. PET film degradation activities of PETase^{WT} and its variants at 30 °C (a, c, e, g & i) and 40 °C (b, d, f, h & j) for 6 days. The released compounds were the sum of MHET and TPA.

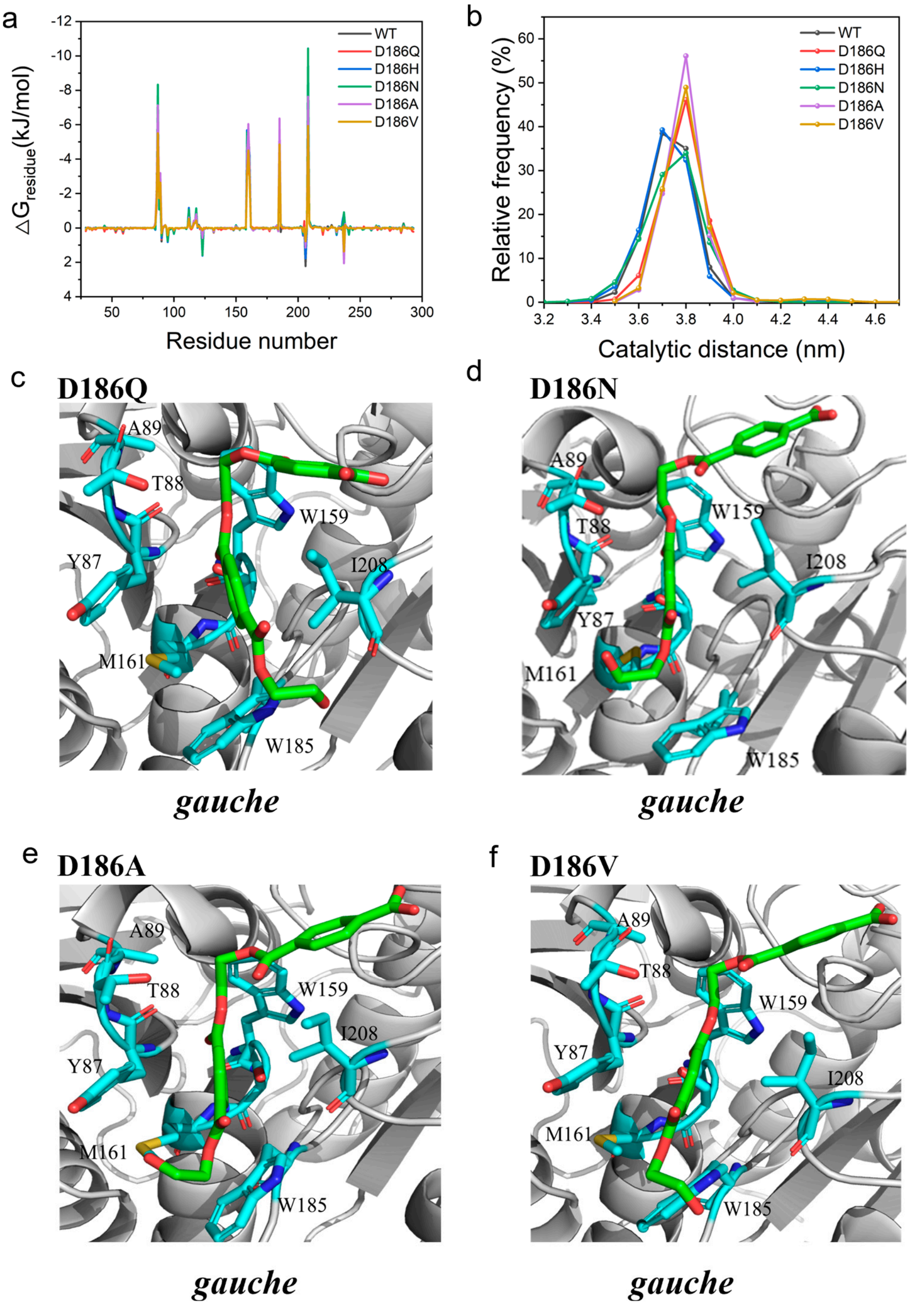

Figure S4. Molecular dynamics (MD) simulations of enzyme-2PET complexes. The binding free energy contribution of each residue (a). The catalytic distances between S160 and 2PET in PETase^{WT} and its variants (b). The binding modes of the substrate 2PET into the active sites of PETase^{D186Q} (c), PETase^{D186N} (d), PETase^{D186A} (e), and PETase^{D186V} (f). The results were generated from six independent MD simulation runs.

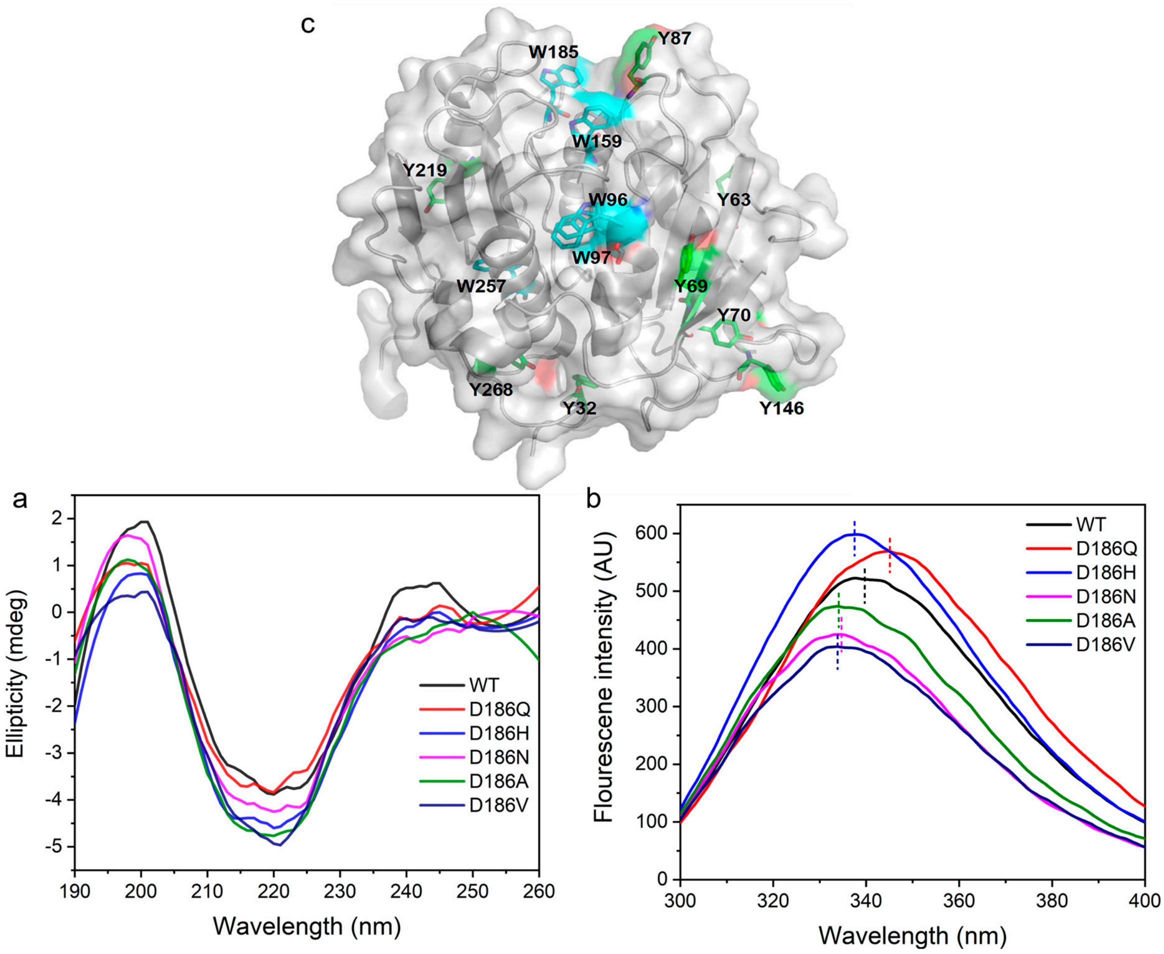

Figure S5. The crystal structure of PETase (PDB ID: 6EQE) (a), where the PETase is shown as the schematic with a surface of 60% transparency and colored grey, the tryptophan residues (W) are shown as the cyan-colored sticks, and the tyrosine residues (Y) are shown as the green-colored sticks. The circular dichroism spectra (b) and the intrinsic fluorescence spectra (c) of $PETase^{WT}$ and its variants at 25 °C.

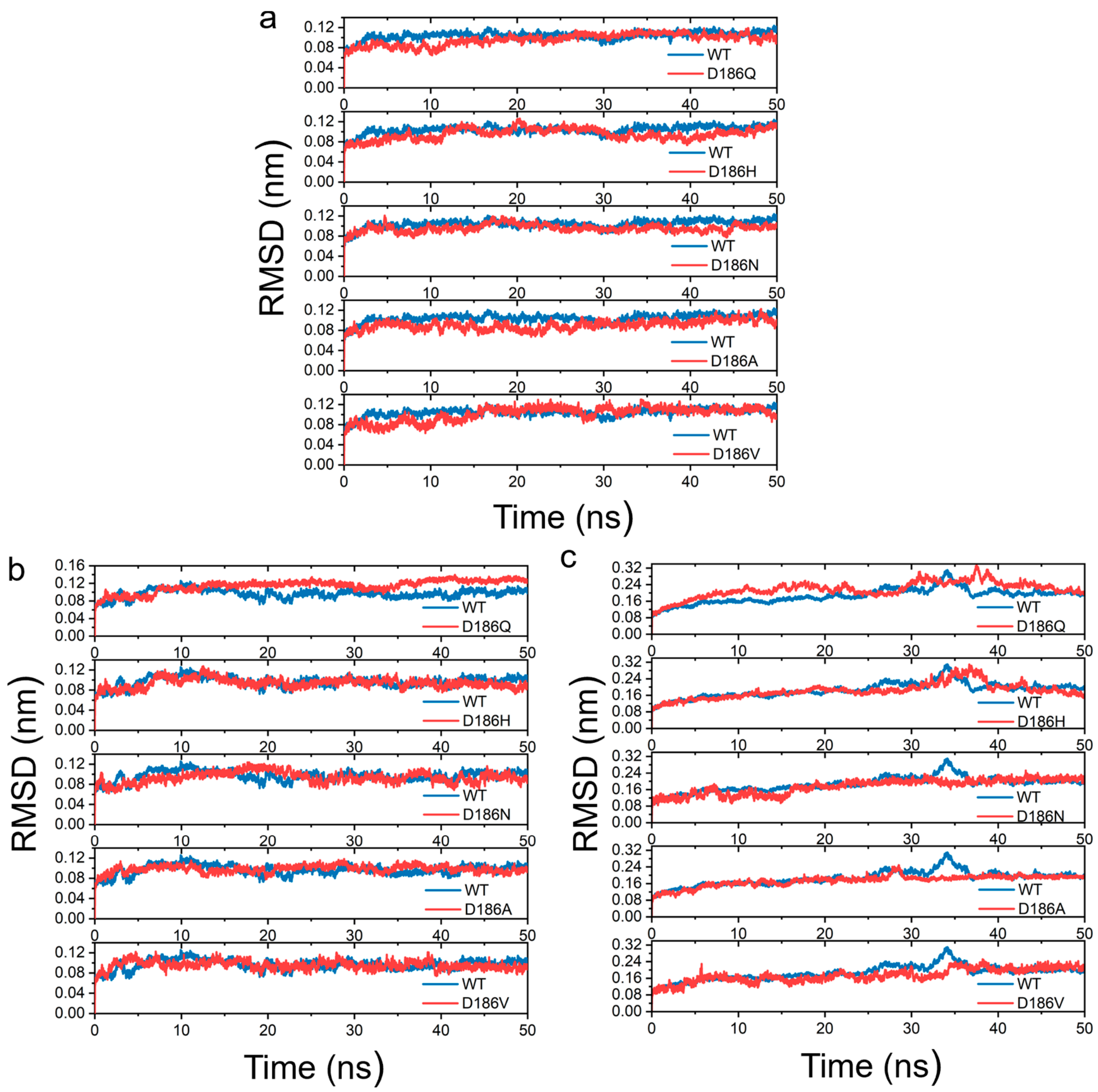

Figure S6. Root mean square deviation (RMSD) of the Cα atoms of PETase^{WT} and its variants during the MD simulations at 303 K (a), 313 K (b), and 403 K (c). The RMSD was calculated from six independent MD simulation runs.

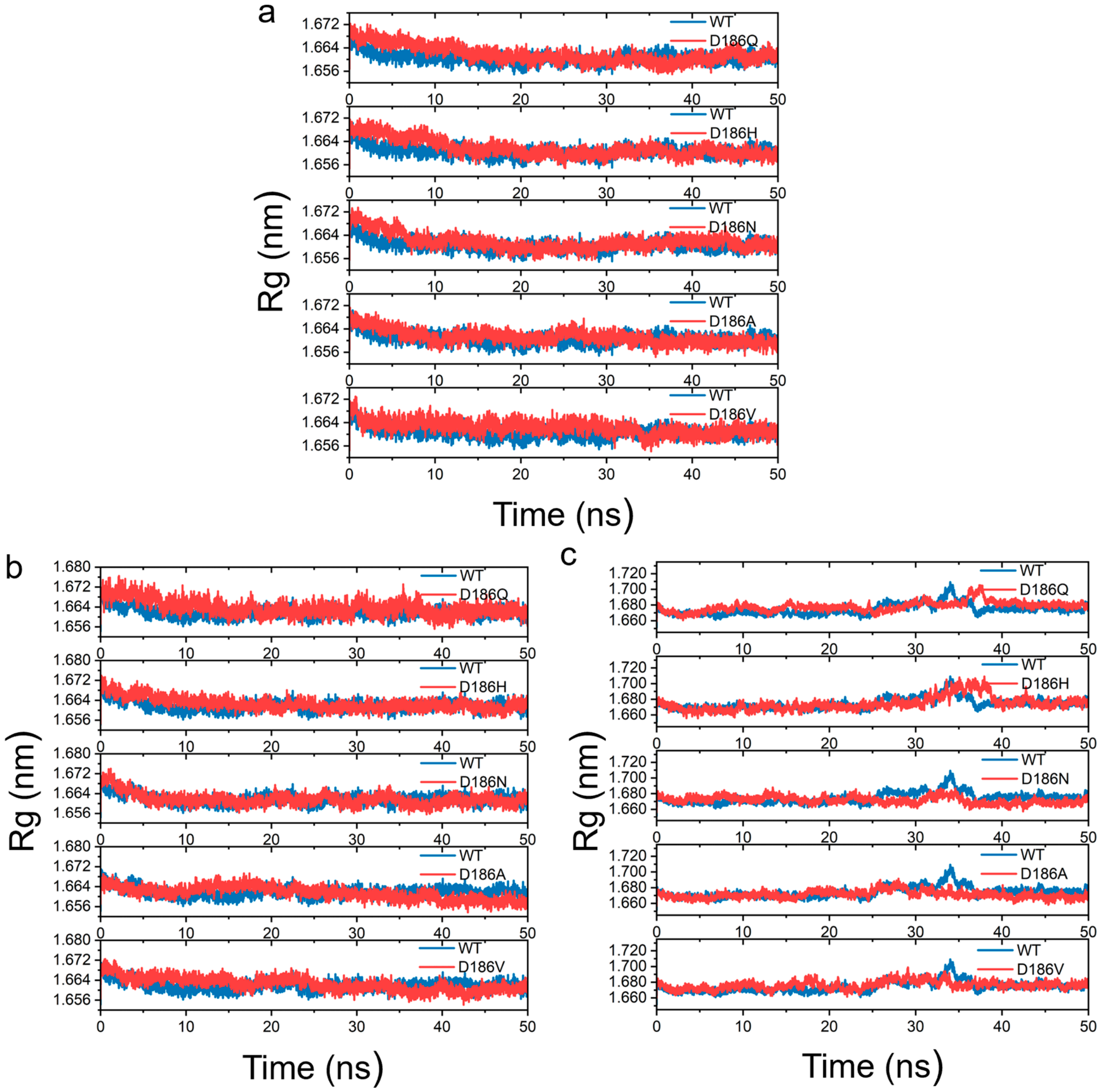

Figure S7. Radius of gyration (R_g) of the Cα atoms of PETase^{WT} and its variants during the MD simulations at 303 K (a), 313 K (b), and 403 K (c). The R_g was calculated from six independent MD simulation runs.

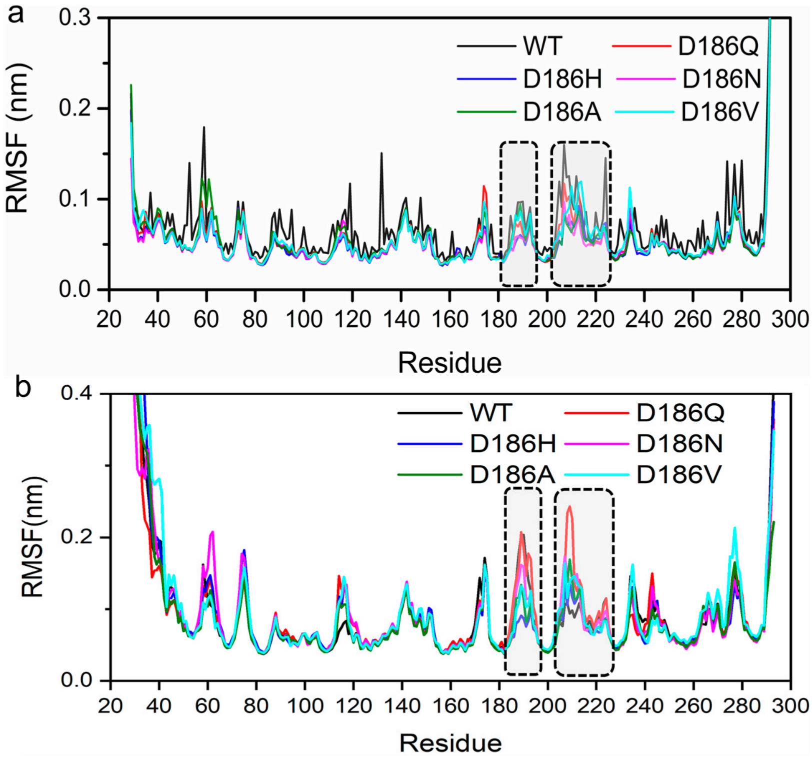

Figure S8. Root mean square fluctuations (RMSF) of the Cα atoms of PETase^{WT} and its variants during the MD simulations at 303 K (a), and 403 K (b), where the regions with large average RMSF differences are marked in dotted boxes. The RMSF was calculated from six independent MD simulation runs.

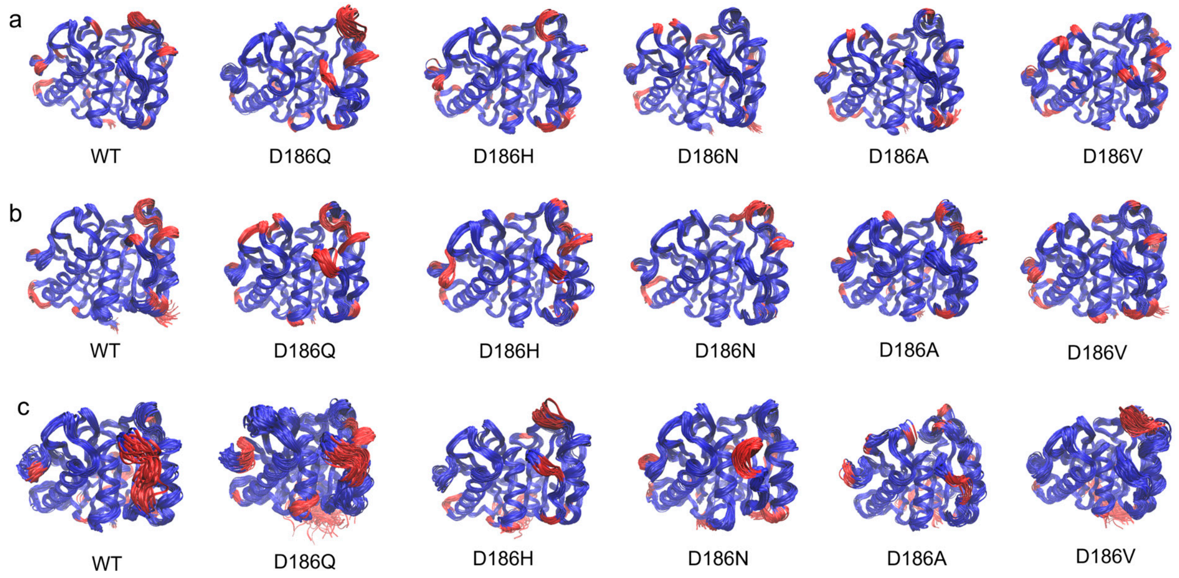

Figure S9. The mobility and structural fluctuations of the Cα atoms of PETase^{WT} and its variants obtained with MDLovofit at 303 K (a), 313 K (b), and 403 K (c). The structural alignment was calculated from the last 10 ns of all trajectories and rendered to illustrate 100 uniformly separated frames. The least mobile Cα atoms are colored in blue and the most mobile atoms are colored in red.

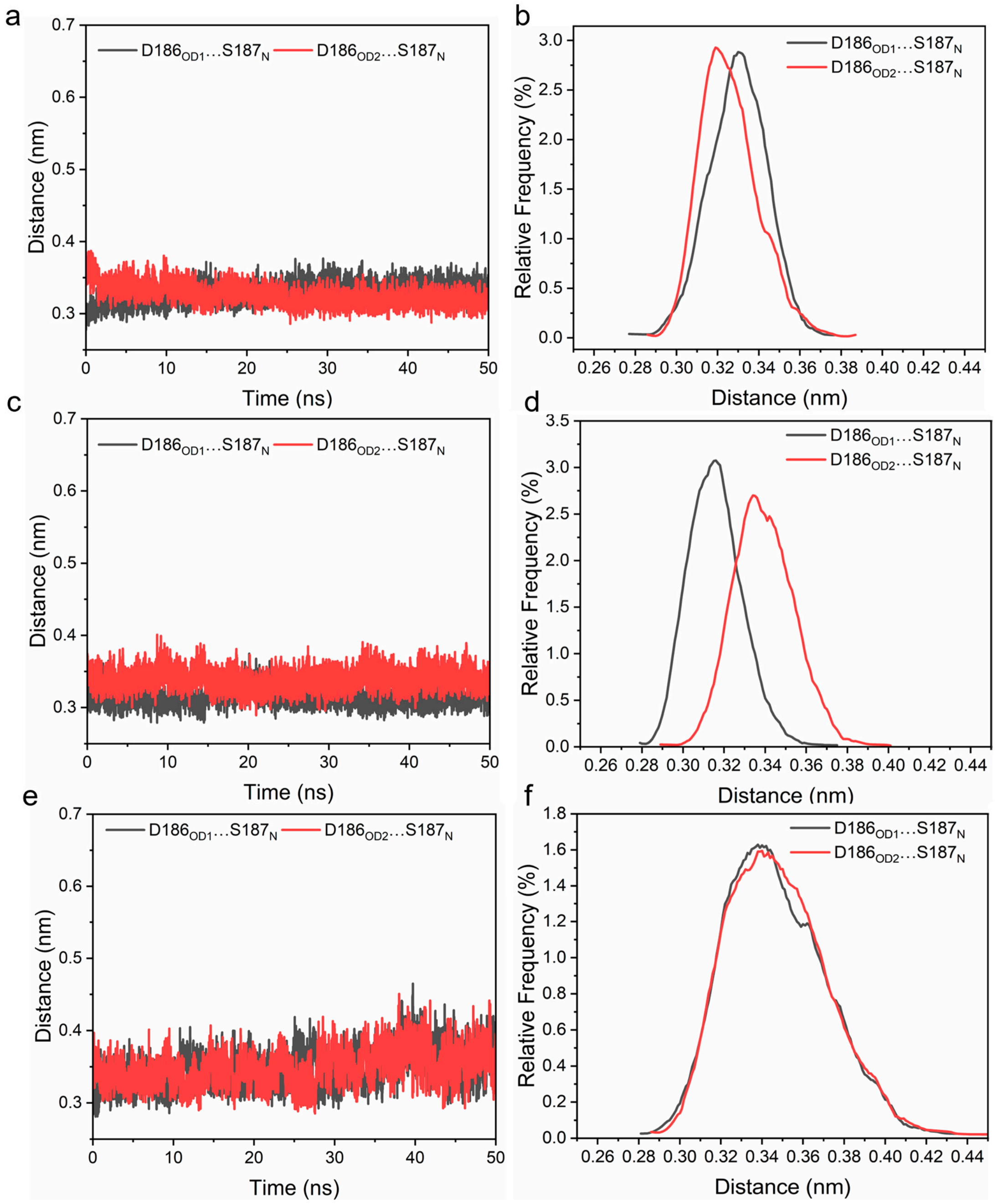

Figure S10. The distance between the atom OD1 or OD2 of D186 and the atom N of S187, and the relative frequency of these two distances in PETase^{WT} during the MD simulations at 303 K (a & b), 313 K (c & d), and 403 K (e & f). The distance was calculated from six independent MD simulation runs.

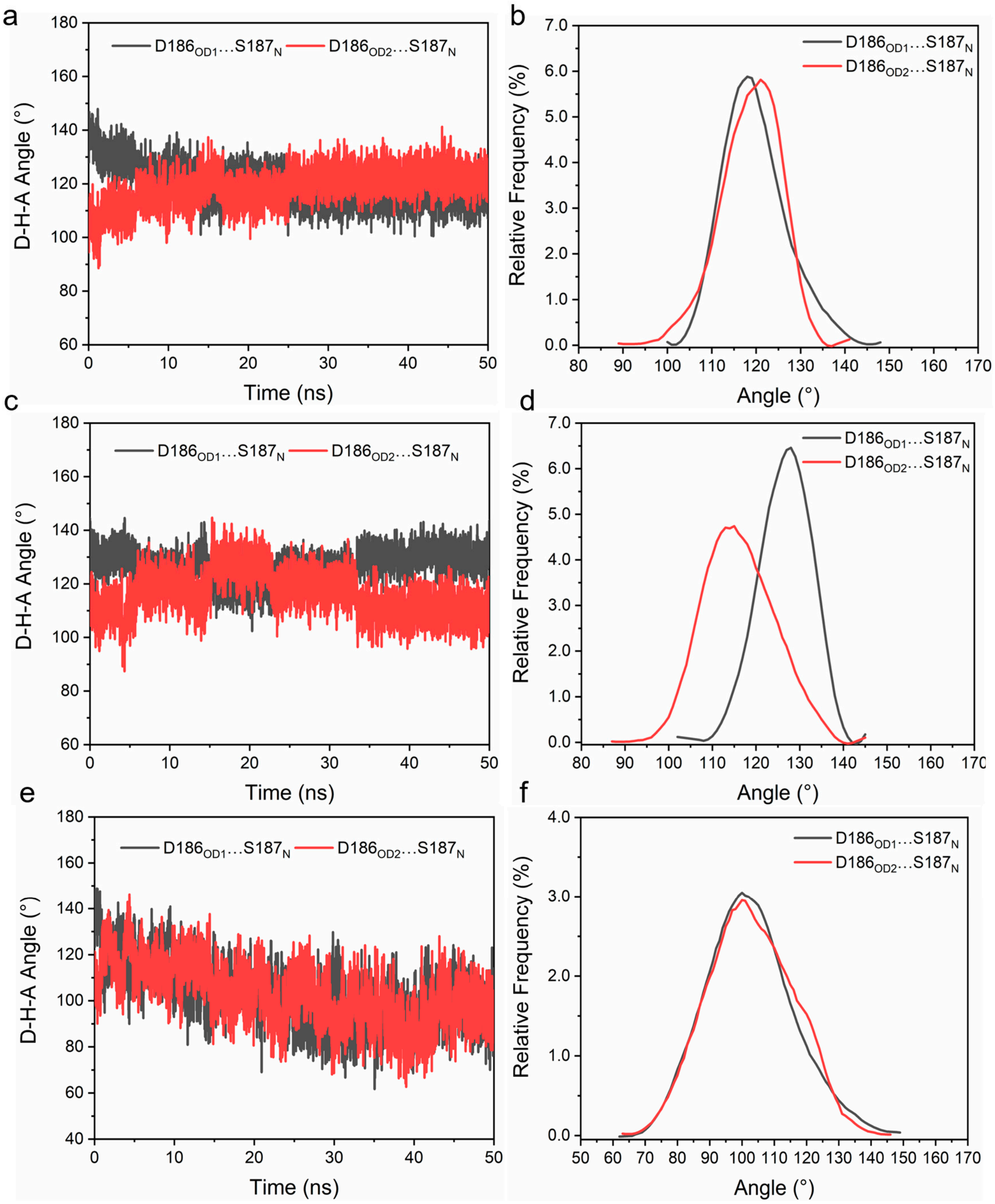

Figure S11. The angle between the acceptor atom (D) of D186OD1 or D186OD2, the hydrogen atom (H), and the donor atom (A) of S187N, and the relative frequency of these two angles in PETase^{WT} during the MD simulations at 303 K (a & b), 313 K (c & d), and 403 K (e & f). The angle constraints were ≥ 90° and ≤ 180°[10], and the angle was calculated from six independent MD simulation runs.

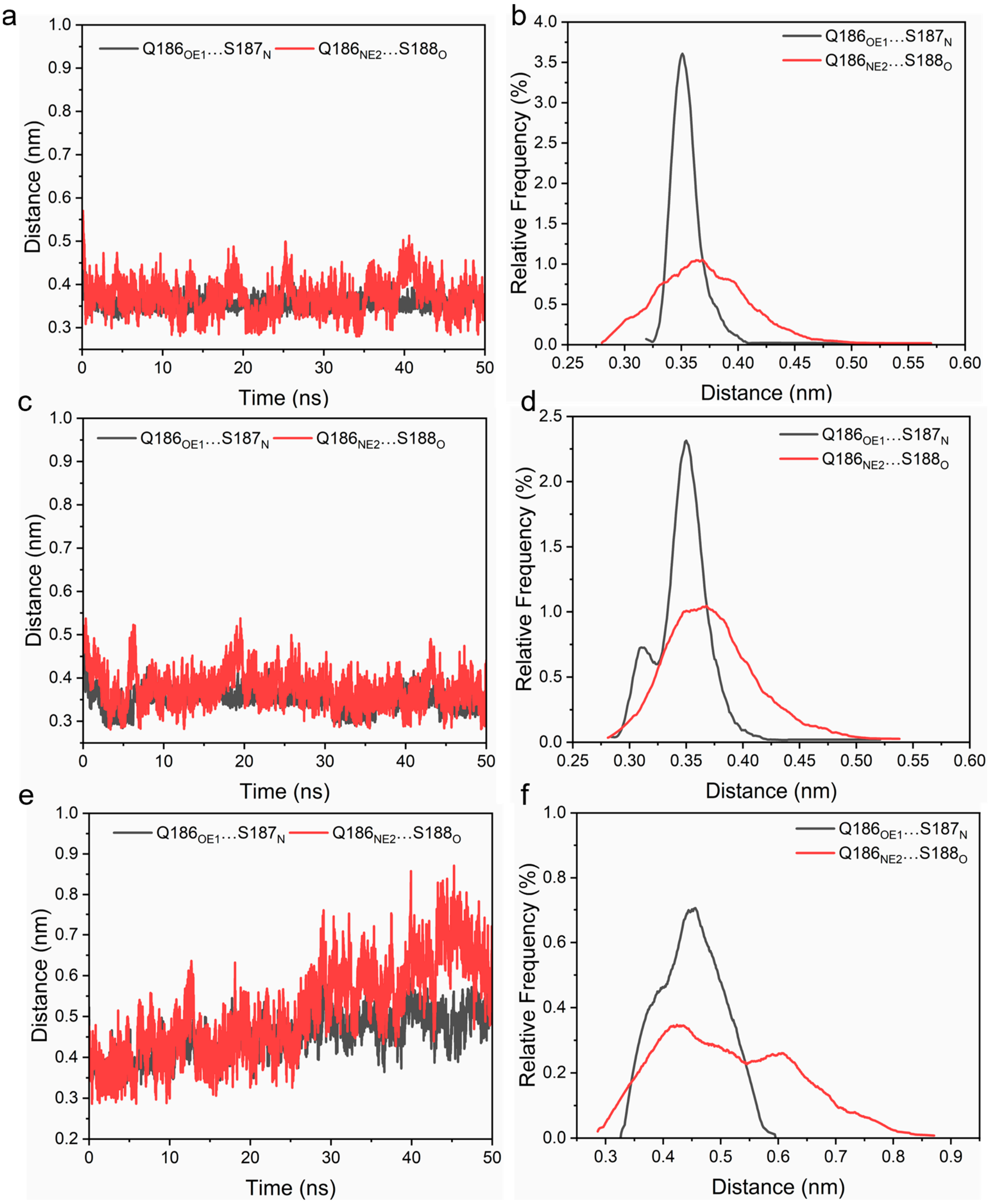

Figure S12. The distance between the atom OE1 of Q186 and the atom N of S187, the distance between the atom NE2 of Q186 and the atom O of S188, and the relative frequency of these two distances in PETase^{D186Q} during the MD simulations at 303 K (a & b), 313 K (c & d), and 403 K (e & f). The distance was calculated from six independent MD simulation runs.

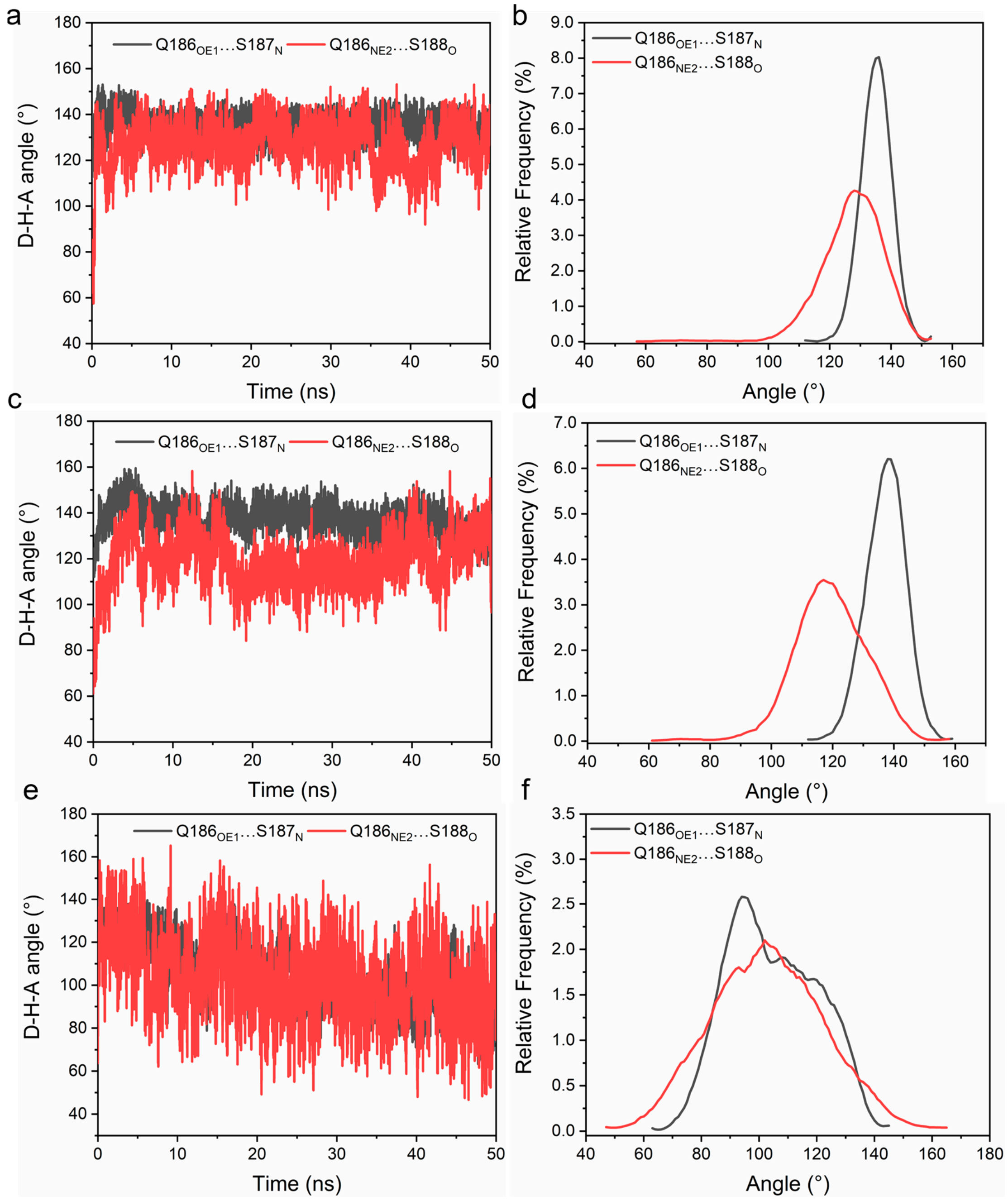

Figure S13. The angle between the the acceptor atom (A) of Q186$_{OE1}$, the hydrogen atom (H), and the donor atom (D) of S187$_{N}$, the angle between the the acceptor atom (A) of S186$_{O}$, the hydrogen atom (H), and the donor atom (D) of S186$_{NE2}$, and the relative frequency of these two angles in PETase^{D186Q} during the MD simulations at 303 K (a & b), 313 K (c & d), and 403 K (e & f). The angle constraints were ≥ 90° and ≤ 180°[10], and the angle was calculated from six independent MD simulation runs.

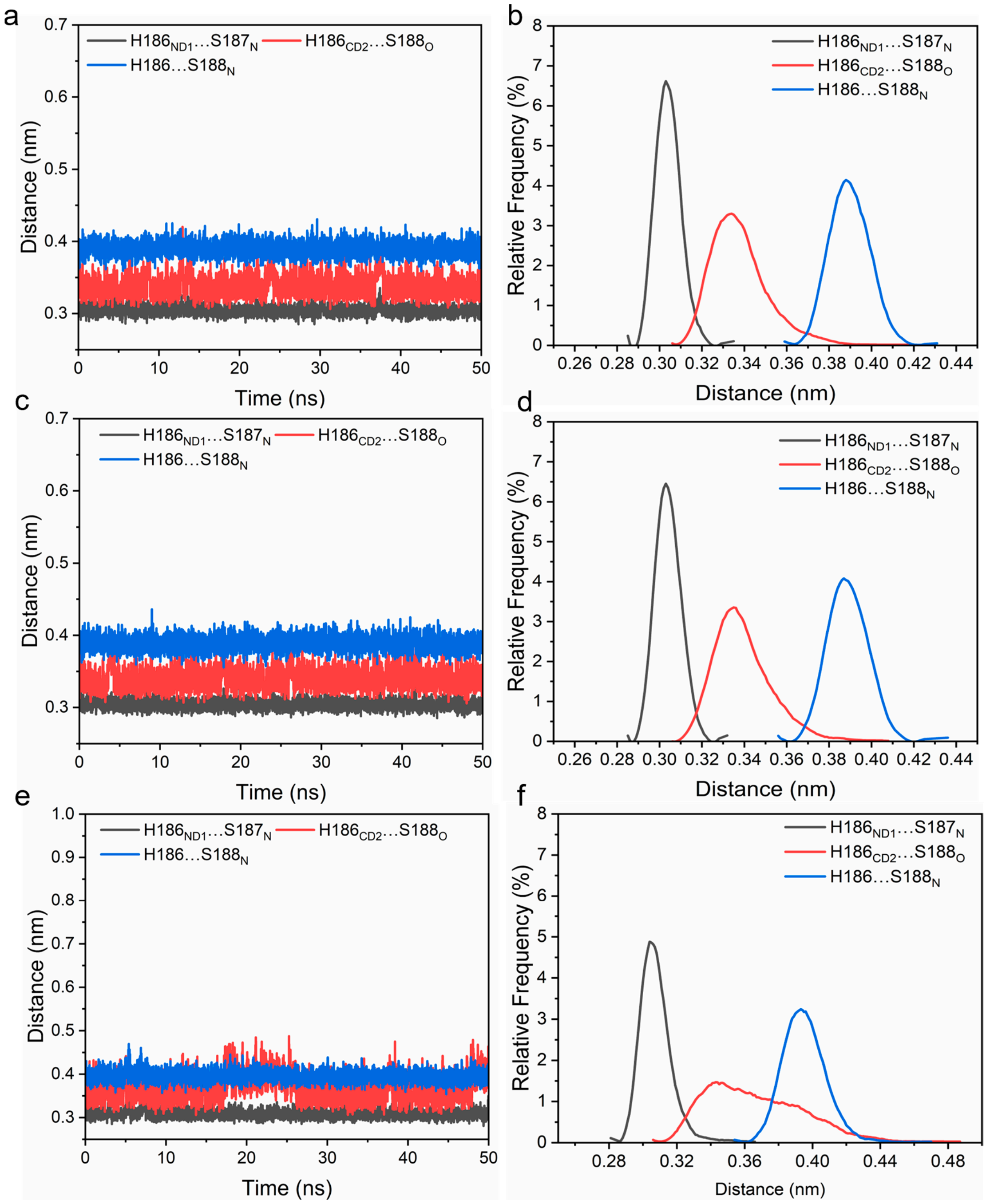

Figure S14. The distance between the atom ND1 of H186 and the atom N of S187, the distance between the atom CD2 of H186 and the atom O of S188, the distance between the centroid of imidazole ring of H186 and the N atom of S188, and the relative frequency of these three distances in PETase^{D186H} during the MD simulations at 303 K (a & b), 313 K (c & d), and 403 K (e & f). The distance was calculated from six independent MD simulation runs.

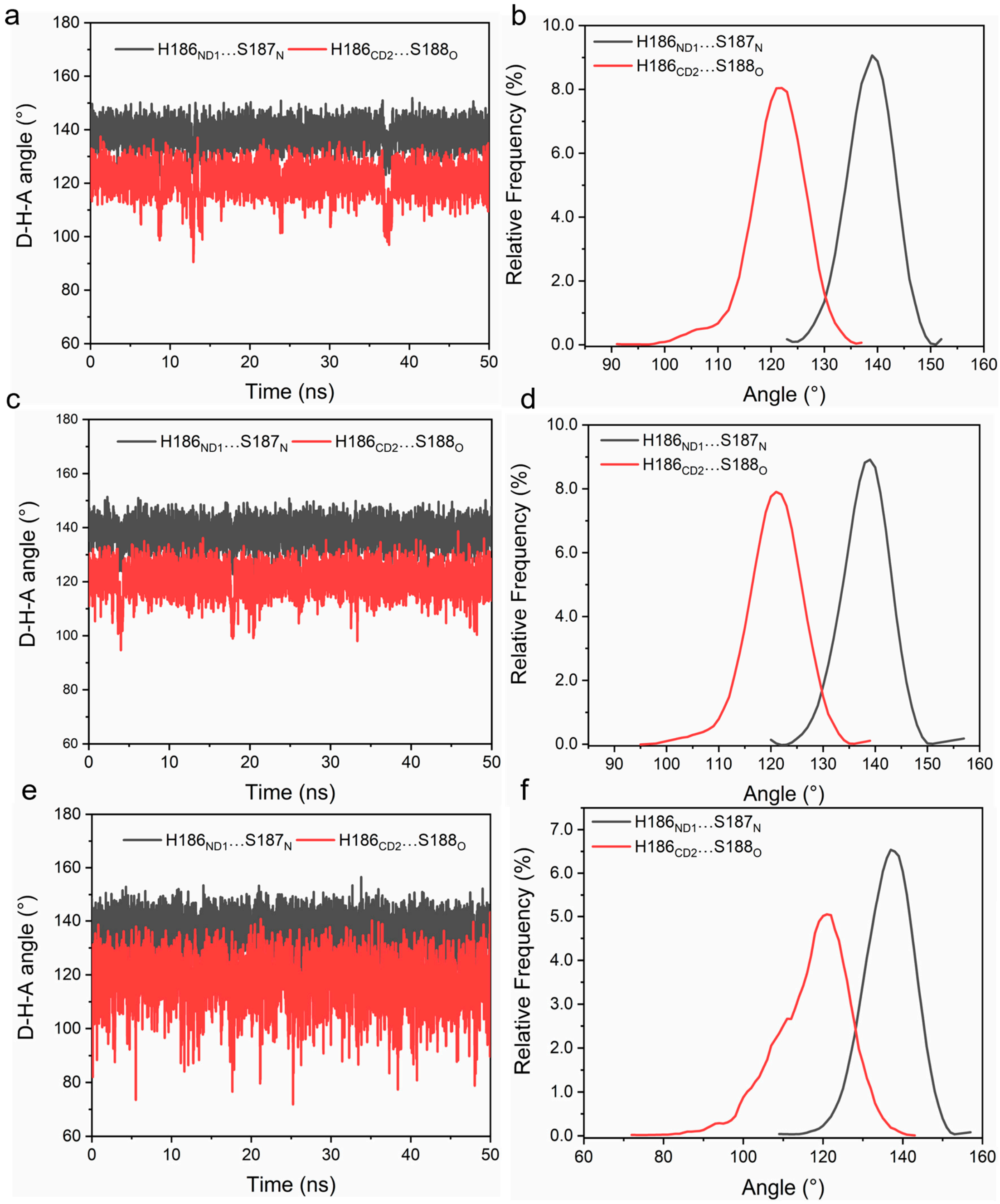

Figure S15. The angle between the acceptor atom (A) of $H186_{ND1}$, the hydrogen atom (H), and the donor atom (D) of $S187_{N}$, the angle between the acceptor atom (A) of $S186_{O}$, the hydrogen atom (H), and the donor atom (D) of $H186_{CD2}$, and the relative frequency of these two angles in $PETase^{D186H}$ during the MD simulations at 303 K (a & b), 313 K (c & d), and 403 K (e & f). The angle constraints were ≥ 90° and ≤ 180°[10], and the angle was calculated from six independent MD simulation runs.

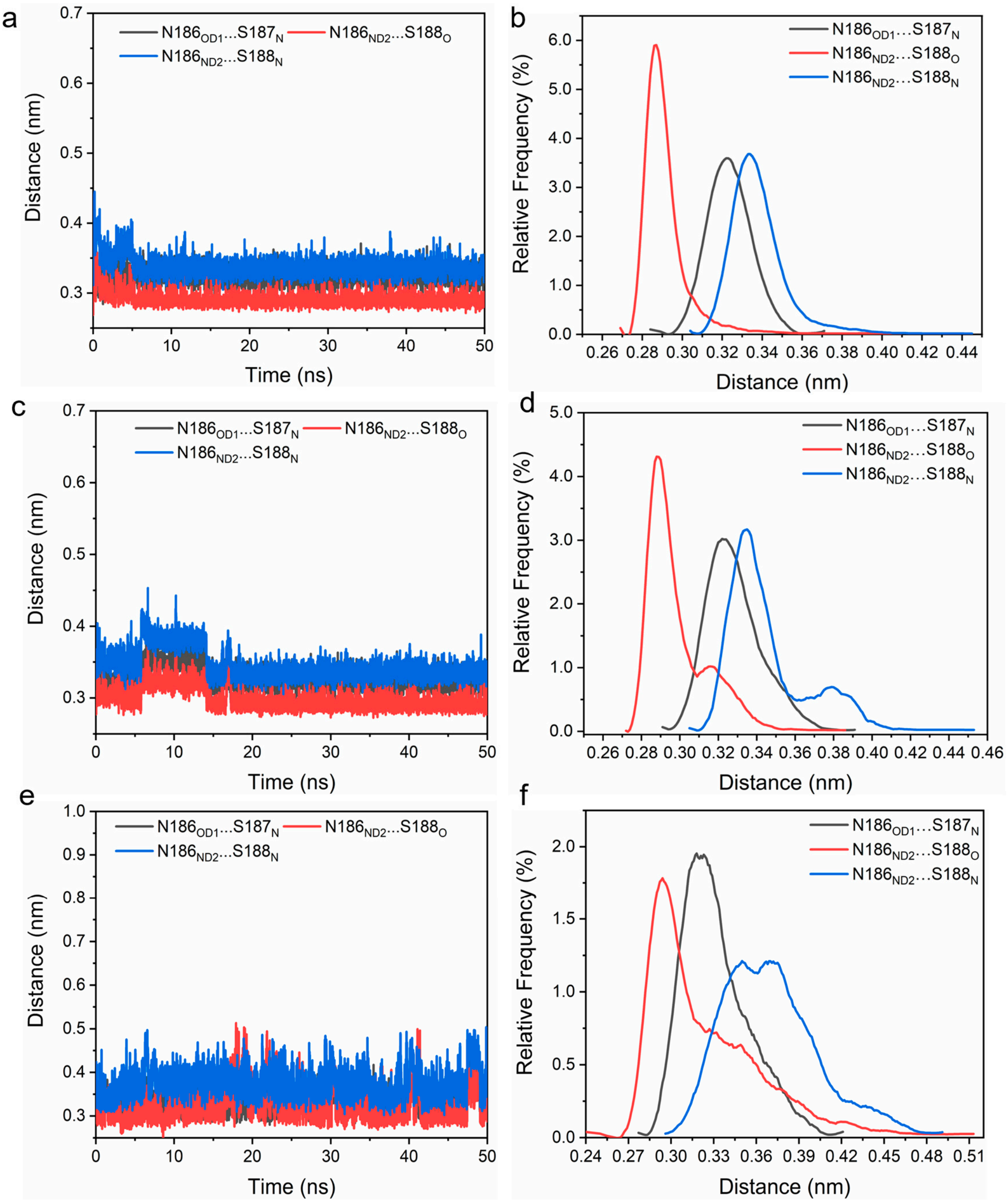

Figure S16. The distance between the atom OD1 of N186 and the atom N of S187, the distance between the atom ND2 of N186 and the atom O of S188, the distance between the atom ND2 of N186 and the atom N of S188, and the relative frequency of these two distances in PETase^{D186N} during the MD simulations at 303 K (a & b), 313 K (c & d), and 403 K (e & f). The distance was calculated from six independent MD simulation runs.

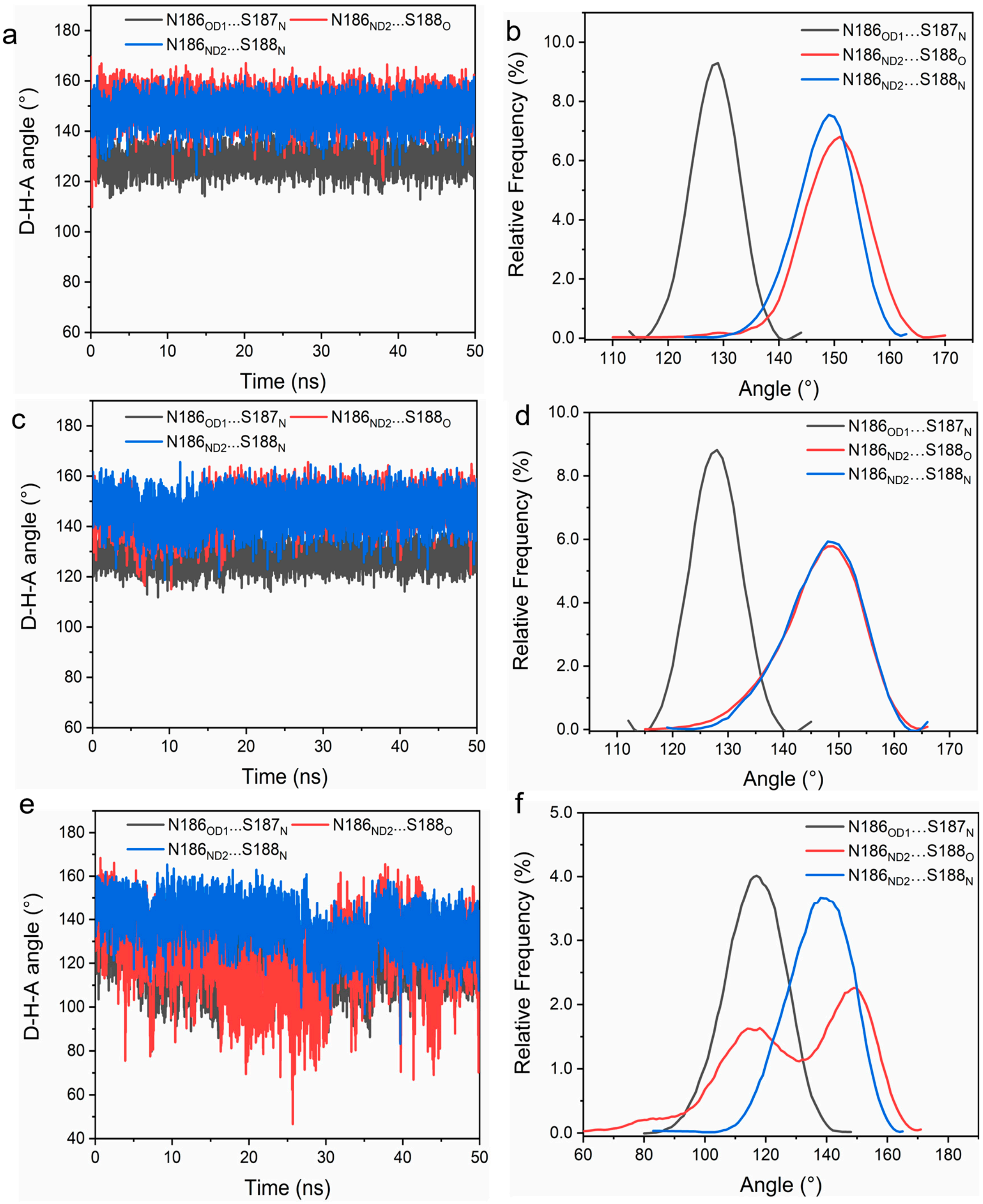

Figure S17. The angle between the acceptor atom (A) of N186$_{OD1}$, the hydrogen atom (H), and the donor atom (D) of S187$_{N}$, the angles between the donor atom (D) of N186$_{ND2}$, the hydrogen atom (H), and the acceptor atom (A) of S188$_{O}$ or S188$_{N}$, and the relative frequency of these three angles in PETase^{D186N} during the MD simulations at 303 K (a & b), 313 K (c & d), and 403 K (e & f). The angle constraints were ≥ 90° and ≤ 180°[10], and the angle was calculated from six independent MD simulation runs.

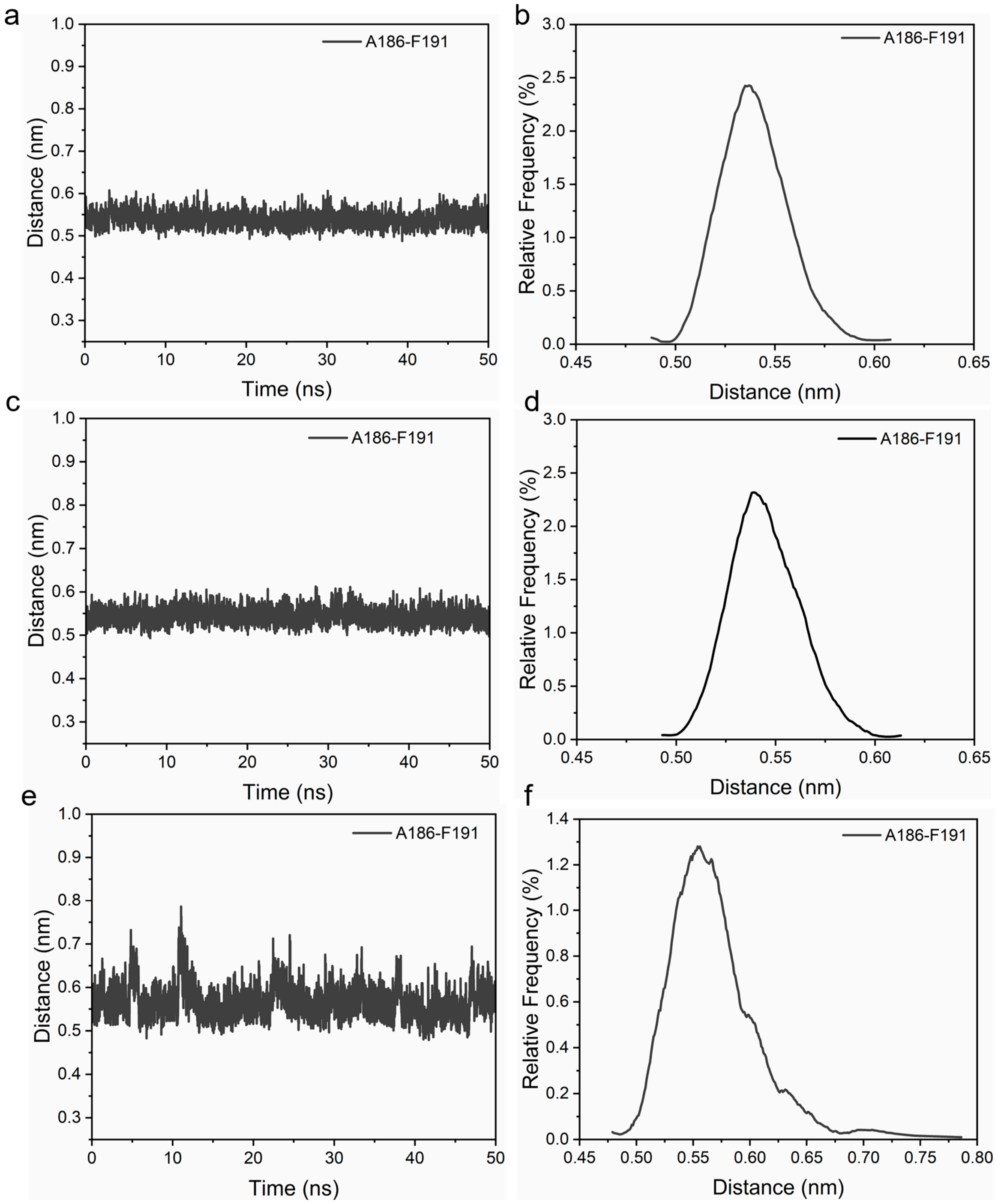

Figure S18. The distance between the centroid of alkyl of A186 and π ring of F191, and the relative frequency of this distance in PETase^{D186A} during the MD simulations at 303 K (a & b), 313 K (c & d), and 403 K (e & f). The distance was calculated from six independent MD simulation runs.

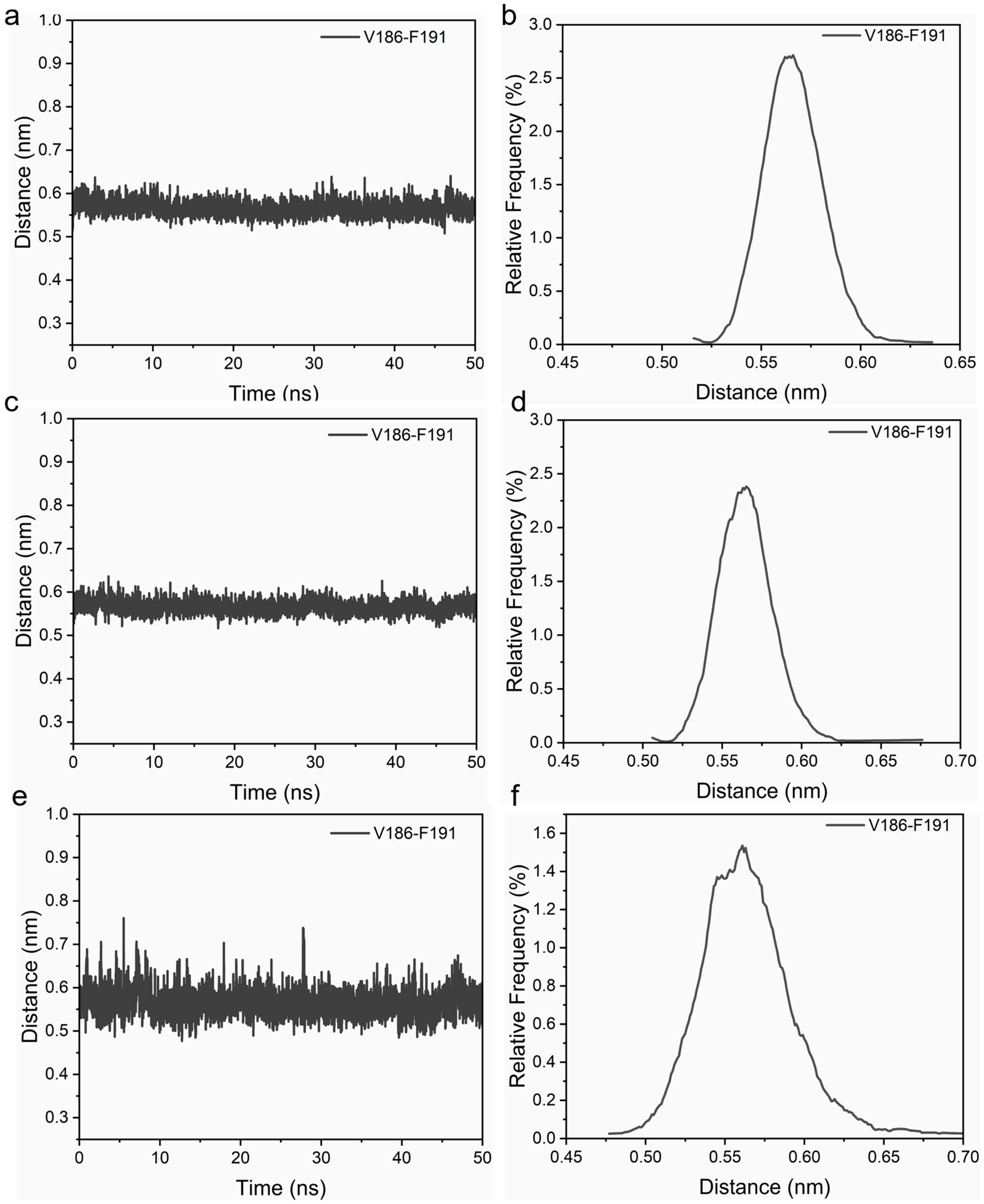

Figure S19. The distance between the centroid of alkyl of V186 and π ring of F191, and the relative frequency of this distance in PETase^{D186V} during the MD simulations at 303 K (a & b), 313 K (c & d), and 403 K (e & f). The distance was calculated from six independent MD simulation runs.

References

1. Meng X.X., Yang L.X., Liu H.Q., Li Q.B., Xu G.S., Zhang Y., Guan F.F., Zhang Y.H., Zhang W., Wu N.F., Tian J. Protein engineering of stable IsPETase for PET plastic degradation by Premuse. *Int J Biol Macromol.* **2021**, 180, 667-676. https://doi.org/10.1016/j.ijbiomac.2021.03.058
2. Son H.F., Cho I.J., Joo S., Seo H., Sagong H.-Y., Choi S.Y., Lee S.Y., Kim K.-J. Rational Protein Engineering of Thermo-Stable PETase from Ideonella sakaiensis for Highly Efficient PET Degradation. *ACS Catal.* **2019**, 9, 3519-3526. https://doi.org/10.1021/acscatal.9b00568
3. Zhong-Johnson E.Z.L., Voigt C.A., Sinskey A.J. An absorbance method for analysis of enzymatic degradation kinetics of poly(ethylene terephthalate) films. *Sci Rep.* **2021**, 11, 2045-2322. https://doi.org/10.1038/s41598-020-79031-5
4. Yin Q.D., You S.P., Zhang J.X., Qi W., Su R.X. Enhancement of the polyethylene terephthalate and mono-(2-hydroxyethyl) terephthalate degradation activity of Ideonella sakaiensis PETase by an electrostatic interaction-based strategy. *Bioresour. Technol.* **2022**, 364, 128026. https://doi.org/10.1016/j.biortech.2022.128026
5. Cui Y.L., Chen Y.C., Liu X.Y., Dong S.J., Tian Y.E., Qiao Y.X., Mitra R., Han J., Li C.L., Han X., Liu W.D., Chen Q., Wei W.Q., Wang X., Du W.B., Tang S.Y., Xiang H., Liu H.Y., Liang Y., Houk K.N., Wu B. Computational Redesign of a PETase for Plastic Biodegradation under Ambient Condition by the GRAPE Strategy. *ACS Catal.* **2021**, 11, 1340-1350. https://doi.org/10.1021/acscatal.0c05126
6. Liu Y.D., Liu Z.Z., Guo Z.Y., Yan T.T., Jin C.X., Wu J. Enhancement of the degradation capacity of IsPETase for PET plastic degradation by protein engineering. *Sci Total Environ.* **2022**, 834, 154947. https://doi.org/10.1016/j.scitotenv.2022.154947
7. Lu H.Y., Diaz D.J., Czarnecki N.J., Zhu C.Z., Kim W.T., Shroff R., Acosta D.J., Alexander B.R., Cole H.O., Zhang Y., Lynd N.A., Ellington A.D., Alper H.S. Machine learning-aided engineering of hydrolases for PET depolymerization. *Nature.* **2022**, 604, 662-667. https://doi.org/10.1038/s41586-022-04599-z
8. Bell E.L., Smithson R., Kilbride S., Foster J., Hardy F.J., Ramachandran S., Tedstone A.A., Haigh S.J., Garforth A.A., Day P.J.R., Levy C., Shaver M.P., Green A.P. Directed evolution of an efficient and thermostable PET depolymerase. *Nat. Catal.* **2022**, 5, 673-681. https://doi.org/10.1038/s41929-022-00821-3
9. Shi L.X., Liu P., Tan Z.J., Zhao W., Gao J.F., Gu Q., Ma H.W., Liu H.F., Zhu L.L. Complete Depolymerization of PET Wastes by an Evolved PET Hydrolase from Directed Evolution. *Angew Chem Int Edit.* **2023**, 62, e202218390. https://doi.org/10.1002/anie.202218390
10. Baker E.N., Hubbard R.E. Hydrogen bonding in globular proteins. *Prog Biophys Mol Biol.* **1984**, 44, 97-179. https://doi.org/10.1016/0079-6107(84)90007-5
